# A new large canopy-dwelling species of *Phyllodytes* Wagler, 1930 (Anura, Hylidae) from the Atlantic Forest of the state of Bahia, Northeastern Brazil

**DOI:** 10.7717/peerj.8642

**Published:** 2020-06-23

**Authors:** Iuri R. Dias, Gabriel Novaes-e-Fagundes, Antonio Mollo Neto, Juliana Zina, Caroline Garcia, Renato Sousa Recoder, Francisco Dal Vechio, Miguel Trefaut Rodrigues, Mirco Solé

**Affiliations:** 1 Programa de Pós-Graduação em Zoologia, Universidade Estadual de Santa Cruz, Ilhéus, Bahia, Brazil; 2 Instituto de Biociências, Universidade de São Paulo, São Paulo, São Paulo, Brazil; 3 Departamento de Ciências Biológicas, Universidade Estadual do Sudoeste da Bahia, Jequié, Bahia, Brazil; 4 Herpetology Section, Zoologisches Forschungsmuseum Alexander Koenig, Bonn, Germany

**Keywords:** Amphibia, Biodiversity, Bromeliad, Integrative taxonomy, Lophyohylini

## Abstract

The known diversity of treefrogs of the genus *Phyllodytes* has rapidly increased in recent years, currently comprising 14 species. Recent field work in the Atlantic Rainforest of the state of Bahia lead to the discovery of a new large species of *Phyllodytes* which is herein described based on multiple evidence including morphological, acoustical and genetic data. *Phyllodytes* sp. nov. is one of the largest species within the genus and presents immaculate yellowish dorsum and limbs. The advertisement call of the species is composed of 7–31 notes (half pulsed/pulsatile-half harmonic) with frequency-modulated harmonics. *Phyllodytes* sp. nov. has a karyotype of 2*n* = 22 chromosomes, as also found in other species of the genus. Genetic distance values of the 16S mitochondrial rRNA among *Phyllodytes* sp. nov. and its congeners range between 6.4 to 10.2%. The description of another new species for this state reinforces the need for further taxonomic work with *Phyllodytes* in this region that has been revealed as a priority area for research and conservation of this genus.

## Introduction

The genus *Phyllodytes* Wagler, 1830 assembles 14 species, distributed mainly throughout the morphoclimatic domain of the Atlantic Forest, most of them with occurrence in the northeast of Brazil (Orrico, Dias & Marciano-Jr, 2018; Frost, 2019;). These small to medium-sized tree frogs (18.2–48.5 mm SVL) are characterized mainly by the presence of odontoids on their mandibles and association with bromeliads, where they complete their entire life cycle (Bokermann, 1966; Peixoto, 1995; Cruz, Feio & Cardoso, 2006).

Although some species of *Phyllodytes* can use terrestrial bromeliads (Ferreira, Schineider & Teixeira, 2012; Cunha & Napoli, 2016), most calling males are heard from bromeliads in the forest canopy, hampering the sampling of representatives of this genus. Thus, this peculiar life history trait may explain the scarcity of studies dealing with its taxonomy, ecology and phylogeny (Marciano-Jr, Lantyer-Silva & Solé, 2017). Fortunately, this scenario is changing due to the greater attention that the genus has received recently, with half of the species within the genus (i.e., seven species) having been described in the last 15 years (Frost, 2019).

The state of Bahia is a diversity hotspot for *Phyllodytes* with nine of the 14 known species (Orrico, Dias & Marciano-Jr, 2018). Notwithstanding, these numbers may underestimate the actual number of species as inventories carried out in coastal areas of the south of the state indicate difficulties in assigning species names to collected individuals (Dias et al., 2014; Dias, Mira-Mendes & Solé, 2014; Mira-Mendes et al., 2018), suggesting that the diversity within the genus may be even larger. This assumption has been corroborated by the description of three new endemic species from southern Bahia in the last three years (Marciano-Jr, Lantyer-Silva & Solé, 2017; Vörös, Dias & Solé, 2017; Orrico, Dias & Marciano-Jr, 2018).

During recent field trips to the Atlantic Forest of southern Bahia we collected specimens of *Phyllodytes* (adults and tadpoles) from both terrestrial and epiphytic bromeliads. A closer examination revealed that these specimens correspond to an undescribed species. Herein, we describe this new species based on morphology of adults and tadpoles, bioacoustics, cytogenetic and molecular evidence. In addition, we compile and discuss available information on bioacoustic features and tadpole morphology of the genus.

## Materials & Methods

### Morphological data

We analyzed specimens housed at herpetological collections of Museu de Zoologia da Universidade Estadual de Santa Cruz, Ilhéus, state of Bahia, Brazil (MZUESC), Museu de História Natural de Jequié, Coleção Herpetológica, Jequié, state of Bahia, Brazil (MHNJCH) and Museu de Zoologia da Universidade de São Paulo, state of São Paulo, Brazil (MZUSP). Examined specimens are listed in Appendix 1. For comparisons with *Phyllodytes brevirostris*, *P. edelmoi* and *P. gyrinaethes*, we used data available in the literature (Peixoto & Cruz, 1988; Peixoto, Caramaschi & Freire, 2003). Specimens collected for this work were obtained under IBAMA #12920-1 and ICMBIO #13708-1 and #35068 permits. This research was approved by the ethics committee on the use of animals (CEUA-UESC 002/12).

We took the following measurements of adult specimens: SVL (snout-vent length), HL (head length), HW (head width), IND (internarial distance), END (eye-nostril distance), ED (eye diameter), IOD (interorbital distance), TD (tympanum diameter), THL (thigh length), TBL (tibia length), TAL (tarsus length), FL (foot length), HAL (hand length), DF3 (width of disc on finger III) and 4TD (toe IV disc diameter). We measured SVL, HL, HW, THL, TL and FL with calipers to the nearest 0.02 mm. The remaining measurements were made with calipers under a stereo microscope. Measurements followed Kok & Kalamandeen (2008) with the exception of IOD that was measured between the anterior corners of the eyes in order to enhance repeatability. Snout profile terminology followed Heyer et al. (1990), texture of dorsal skin is described according to Kok & Kalamandeen (2008) and webbing formula notation followed Savage & Heyer (1967) and Savage & Heyer (1997), measured on left hand and foot. Colour in life was described based on photographs of live specimens taken during the day.

### Cranial osteology

We scanned two individuals of the new species, one adult (MZUSP 157524) and one juvenile (MZUSP 157526) with a SkyScan digital microtomograph with a resolution of 18 µm and of 9 µm, respectively. The images were processed in CT Analyzer v.1.11 software. The resulting 3D models were visualized on CTvox v.3.3 software, and the osteological descriptions were made based on three-dimensional images. Terminology of osteological elements follows Trueb (1973) and Duellmann & Trueb (1986).

### Tadpole

Two tadpoles (MZUSP 157525) of the new species were obtained together with an adult male (MZUSP 157524), in a large bromeliad (ca. 100 cm diameter) on a trunk of a large tree at 8 m height at Estação Ecológica Estadual de Wenceslau Guimarães, Wenceslau Guimarães municipality, State of Bahia, Brazil (13°35′41.8″S, 39°43′10.5″W). Mitochondrial sequences obtained from a piece of the tail fin of one tadpole at stage 27 of Gosner (1960) confirmed their conspecificity (tadpoles obtained with MTR 22178 referred to as *Phyllodytes* sp. 2 in Blotto et al., 2020). Tadpoles were euthanized in 5% lidocaine, fixed and preserved in 10% formalin. Terminology for external morphology follows Altig & McDiarmid (1999). Tadpole description and illustrations are based on the specimen at developmental stage 35 of Gosner (1960), and compared with the specimen at stage 27.

We measured 13 morphometric variables with a Digimess^®^ digital caliper (precision ± 0.01 mm) and a micrometer ocular in a Nikon SMZ645 stereomicroscope following Altig & McDiarmid (1999): total length (TL), body length (BL), body height (BH), body width (BW), interorbital distance (IOD), internarial distance (IND), eye diameter (ED), eye-nare distance (END), nare-snout distance (NSD), tail length (TAL), maximum tail height (MTH), tail muscle height (TMH) and tail muscle width (TMW). The drawings were prepared with the aid of digital photograph obtained with an Olympus DP72 digital camera attached to an Olympus SZX12 stereomicroscope.

### Bioacoustic data

We recorded the advertisement call of two individuals at their natural habitat before collecting. The holotype was recorded with a digital recorder (Tascam DR1) with a directional microphone (Sennheiser ME45), on March 2nd 2015, between 00:40 am–02:00 am, at a distance of ∼0.5 m and air temperature of 21.9 °C. The animal was calling from a terrestrial bromeliad and no conspecifics were heard nearby. For the analysis of notes, we used only the notes from 10 randomly selected calls.

One paratype (MZUESC 18265) was recorded with a digital recorder (Marantz PMD660) with a shotgun microphone (Sennheiser-ME66), on June 18th 2014, around 00:10 am, at a distance of ∼1 m and air temperature of around 19 °C. The animal was calling from an isolated epiphytic bromeliad (*Hohenbergia* sp.) around 3.1 m above the ground. During the recording, we imitated the frog call (“vocal playback”) to stimulate its response. For this reason, we did not measure the interval between calls, as they would be uninformative. In eight occasions the male emitted a different type of call, which we named “call type II”. We deposited all recordings at the Fonoteca Neotropical Jacques Vielliard (FNJV 40997, 41382–41384).

All callings were recorded in uncompressed PCM format with a sample rate of 44.1 kHz and 16 bits. Sound analysis was made with Raven Pro 1.4. Time components were measured from the waveform, while spectral components were measured from the spectrogram. We set the spectrographic parameters as following: window type: Hann, window size: 1024 samples, 3dB filter bandwidth: 61.9 Hz, time grid overlap: 90%, time grid size: 102 samples, frequency grid DFT size: 1024 samples, frequency grid spacing: 43.1 Hz. When necessary, we filtered the frequencies below 150 Hz to reduce wind sound interference, and above 5000 Hz, to reduce insect sound interference, using the “Filter out active selection” tool. Categorization of sound types follows Beeman (1998), modified by Köhler et al. (2017). We used a note-centered terminology (*sensu* Köhler et al., 2017) for the call elements nomenclature.

The following parameters were measured from the calls: call duration (from peak amplitude of the first note to the peak of the last note of each call), interval between calls (from peak amplitude of the last note of a call to the peak amplitude of the first note of next call) and number of notes of the call. For the analysis of the notes we delimited the selection borders of each note using the threshold of 5% of the maximum amplitude of the note (see Littlejohn, 2001). Based on this selection, the following automated measurements (see Charif, Waack & Strickman, 2010) were taken: duration 90%, dominant frequency, bandwidth 90%, frequency 5% and frequency 95%. Then, we made additional smaller selections to measure the initial and final frequency of the first harmonic (the peak frequency of the first 0.015 s and of the last 0.015 s of the first harmonic). Finally, we measured the inter-note interval, note emission rate and note shape (note rise time divided by note duration).

For the comparisons with the calls of the congeneric species, we used the data available in the published call descriptions. Based on the literature and our personal observations, we assume functional homology between the herein defined notes and those described for other species of the genus *Phyllodytes*.

### Cytogenetic data

We karyotyped one paratype (MZUESC 18265) which was injected with colchicine 0.5% six hours before euthanasia with a lethal dose of lidocaine. Mitotic chromosomes were obtained from intestinal epithelium after 30 min under distilled water and 24 h in cold Carnoy fixer (3:1) following a modified protocol from King & Rofe (1976).

Chromosomic classification followed Levan, Fredga & Sandberg (1964) by adopting the following limits for the Arm Ratio (AR): AR = 1.00 to 1.70, metacentric, AR = 1.71–3.00, submetacentric; AR = 3.01 –7.00 subtelocentric; AR = greater than 7.00, acrocentric. They were arranged in decreasing order of size. Metacentric, submetacentric and subtelocentric chromosomes have two arms, the acrocentric ones a single one.

The C-banding was obtained as described by Sumner (1972) with the following modifications: the blades were immersed in HCl 0.2 N for 13 min at room temperature and then immersed in Ba(OH)_2_ solution at 60 °C for 35 s and in 2X SSC at 60 °C for 30 min. Nucleolar organizing regions (NORs) were identified by silver nitrate impregnation following Howell & Black (1980) and FISH with 18S rDNA probe according to Pinkel, Straume & Gray (1986) and Hatanaka & Galetti (2004).

At least 30 metaphase spreads were analyzed to confirm the 2n and karyotype structure results. Images were captured using an Olympus BX50 microscope (Olympus Corporation, Ishikawa, Japan) with CoolSNAP camera and the images processed using Image Pro Plus 4.1 software (Media Cybernetics, Silver Spring, MD, United States).

### Molecular data

We sequenced the final part of fragment of the mitochondrial 16S ribosomal RNA gene (16S rRNA) from the holotype (MZUESC 18264) and from a paratype (MZUESC 18265). We also sequenced the same fragment for one topotype of *Phyllodytes kautskyi* (Museu de Biologia Mello Leitão - MBML 8818) from Domingos Martins, State of Espírito Santo (GenBank: MN648397 –MN648399; Document S1) following protocols of Faivovich et al. (2005) and using the primers 16SC (F): 5′-GTRGGCCTAAAAGCAGCCAC-3′(Darst & Cannatella, 2004) and 16SBr-H (R): 5′-CCGGTCTGAACTCAGATCACGT-3′(Palumbi et al., 2001).

### Phylogenetic analyses

Sequence alignment was carried out using the MAFFT algorithm with L-INS-i strategy (Katoh & Toh, 2008). Phylogenetic reconstruction was performed using the Bayesian inference with MrBayes 3.2.3 (Huelsenbeck & Ronquist, 2001) after the best model of evolution was determined using jModelTest 2.1.3 (Darriba et al., 2012) based on Akaike information criterion to be GTR+I+G. Bayesian analyses included two independent runs, each with four chains and sampling every 1000 generations for 70 million generations. We examined trace plots and effective sample size in Tracer 1.7 (Rambaut et al., 2018) to check MCMC mixing and convergence. We removed trees from the first 20% of the samples as burn-in. A consensus of the post burn-in trees was visualized in FigTree 1.4.2 (http://tree.bio.ed.ac.uk/software/figtree). These analyses were performed at CIPRES Science Gateway (Miller, Pfeiffer & Schwartz, 2010). We included homologous sequences available in Genbank of all species of *Phyllodytes* and of at least one species from all genera of Lophyohylini tribe (*sensu* Faivovich et al., 2005). We used Cophomantini *Nesorohyla kanaima* to root the tree following the results of a previous phylogenetic analysis (Faivovich et al., 2005; Duellman, Marion & Hedges, 2016). To estimate the genetic distance, we calculated uncorrected p-distances among the species of *Phyllodytes* in MEGA 6.06 (Tamura et al., 2013), considering d:transitions + transversions, uniform rates among sites, and gaps/missing data as complete deletion.

### Nomenclatural acts

The electronic version of this article in Portable Document Format (PDF) will represent a published work according to the International Commission on Zoological Nomenclature (ICZN), and hence the new names contained in the electronic version are effectively published under that Code from the electronic edition alone. This published work and the nomenclatural acts it contains have been registered in ZooBank, the online registration system for the ICZN. The ZooBank LSIDs (Life Science Identifiers) can be resolved and the associated information viewed through any standard web browser by appending the LSID to the prefix http://zoobank.org/. The LSID for this publication is as follows: urn:lsid:zoobank.org:pub:FD2CACD2-F59F-4B75-947D-B576701424D9. The online version of this work is archived and available from the following digital repositories: PeerJ, PubMed Central and CLOCKSS.

## Results

**Generic placement**. **—** The new species can be allocated to the genus *Phyllodytes* by the presence of odontoids in the mandible and phylogenetic reconstructions indicate close relationship to other species in this genus also supporting this placement (see below).

### *Phyllodytes magnus* sp. nov.

**Table:** 

*Phyllodytes kautskyi*—(Freitas, 2015)
*Phyllodytes cf. kautskyi*—(Freitas et al., 2018)
urn:lsid:zoobank.org:act:D8DF9024-57C1-413B-9D05-B0CF05B7929C

**Figure 1:**
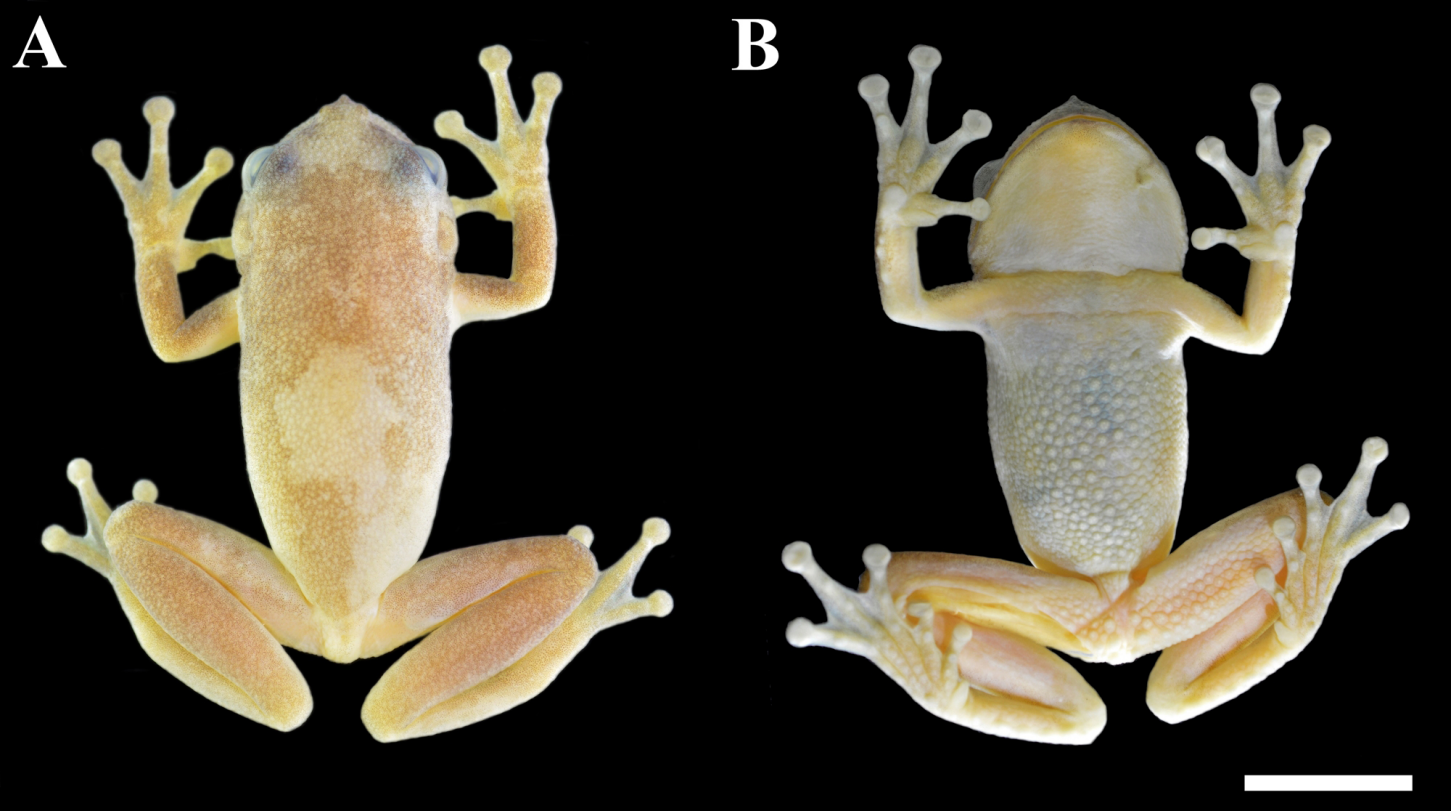
Holotype of *Phyllodytes magnus* sp. nov. (MZUESC 18264; SVL 36.4 mm). (A) Dorsal and (B) ventral view of the body. Scale bar = 10 mm.

**Figure 2:**
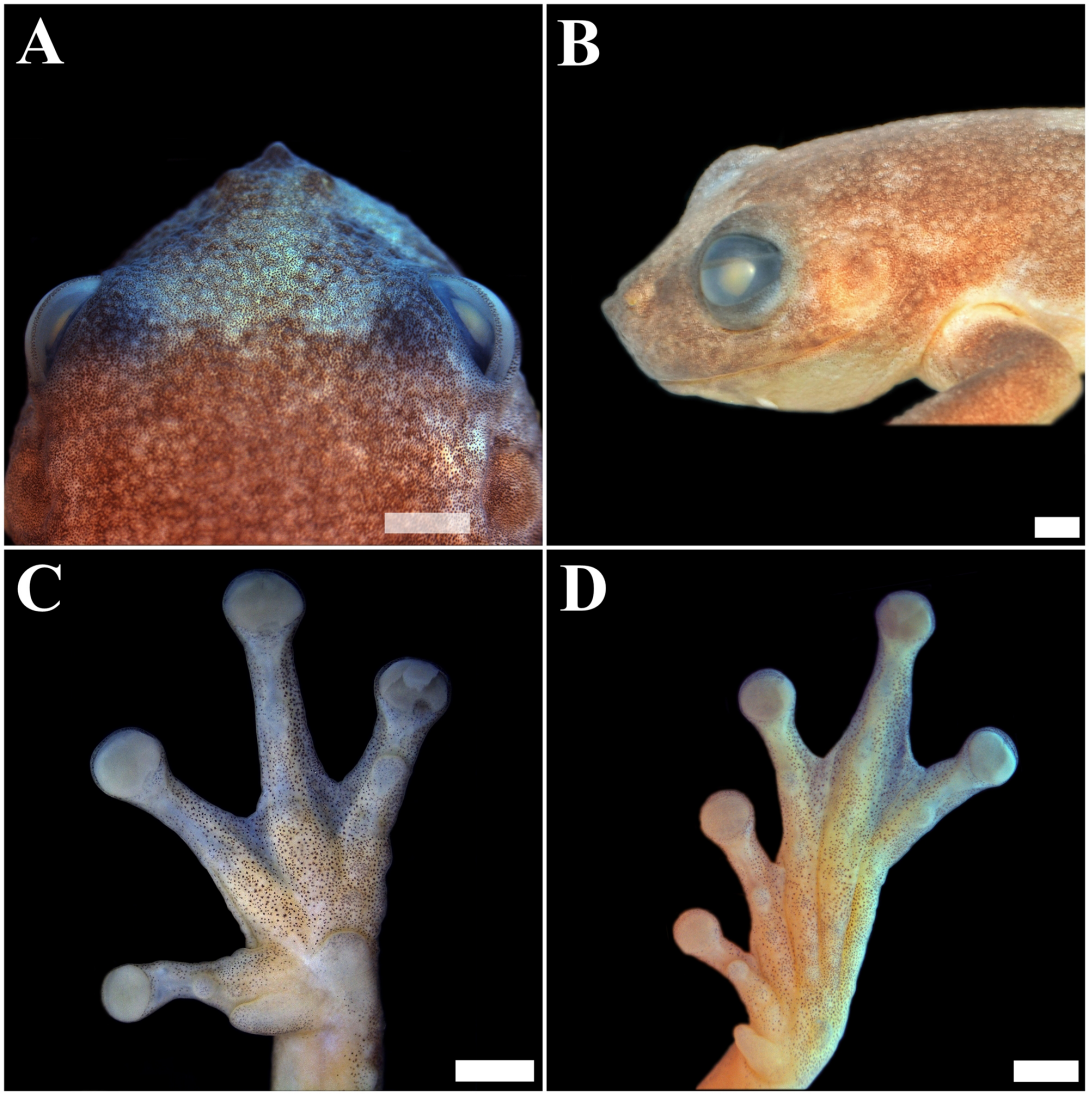
Holotype of *Phyllodytes magnus* sp. nov. (MZUESC 18264). (A) Dorsal view of the head; (B) Lateral view of the head (left side); (C) Plantar view of the left hand and (D) Plantar view of the left foot. Scale bar = two mm.

**Figure 3:**
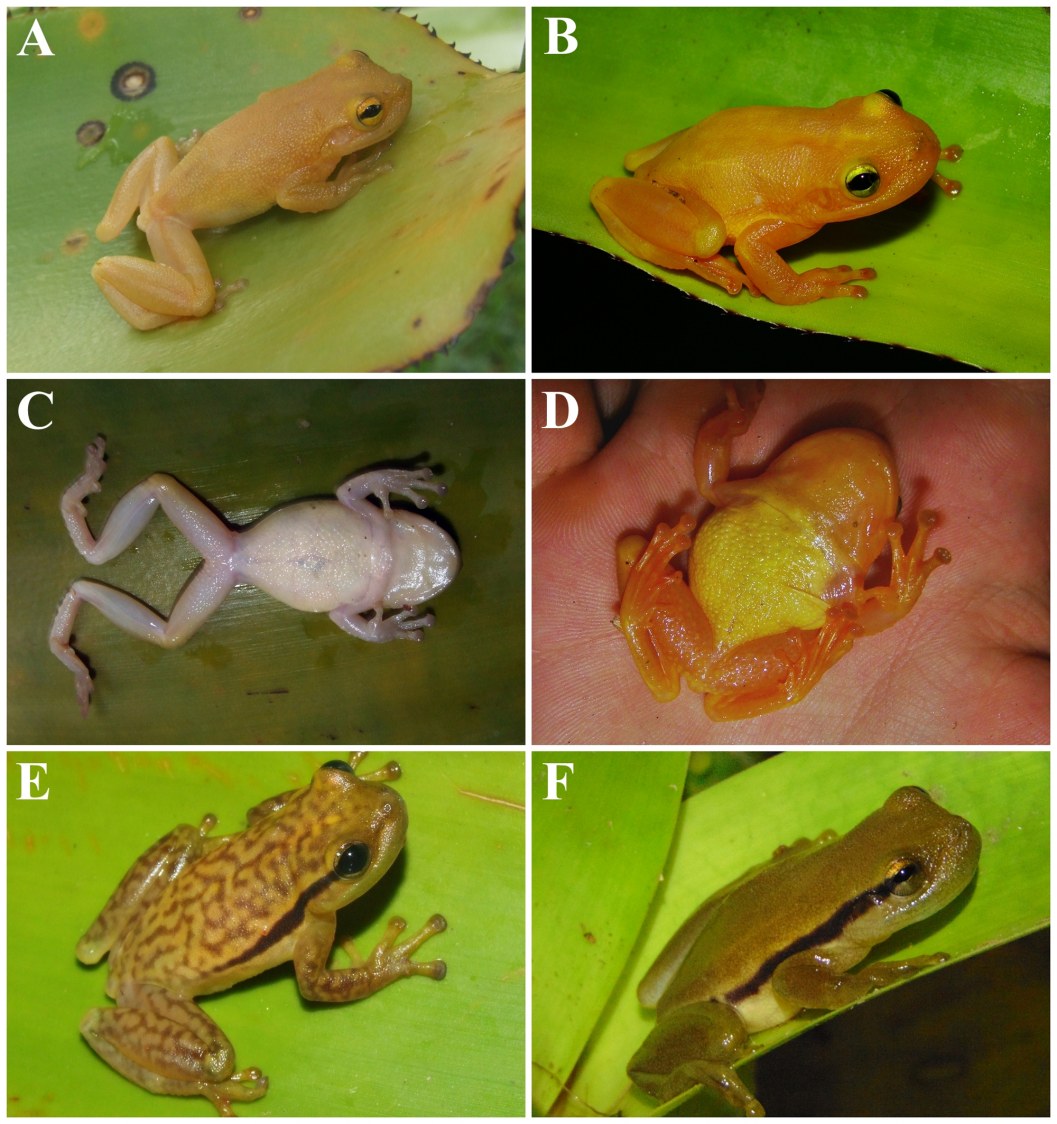
*Phyllodytes magnus* sp. nov (A, B, C, D), *P. maculosus* (E) and *P. kautskyi* (F) in life. *Phyllodytes magnus* sp. nov, holotype (MZUESC 18264) in (A) dorsal and (C) ventral view and paratype (MZUESC 18265) in (B) dorsal and (D) ventral view. In (E) *P. maculosus* and (F) *P. kautskyi*.

**Holotype**. **—** MZUESC 18264, adult male (Figs. 1, 2, 3A and 3C) found in a terrestrial bromeliad, on March 2nd 2015, by IRD and Carlos Augusto Souza-Costa at Serra da Jibóia (12°52′19.08″S, 39°28′53.90″W; 490 m a.s.l.; WGS84), between the municipalities of Santa Terezinha and Elísio Medrado, State of Bahia, Brazil.

**Paratypes**. **—** MZUESC 18265 (ex-MHNJCH 914), adult male (Fig. 3B and 3D), collected in an epiphytic giant bromeliad (*Hohenbergia* sp.) at ∼3.10 m above the ground at Parque Estadual da Serra do Conduru (14°29′36.80″S, 39°08′10.70″W; 201 m a.s.l.; WGS84), municipality of Uruçuca, State of Bahia, Brazil, on June 19th 2014, by GNF and Joedison dos Santos Rocha; MZUSP 157524 (adult male) and MZUSP 157526 (juvenile), both collected in Estação Ecológica Estadual de Wenceslau Guimarães (13°35′41.8″S, 39°43′10.5″W; 530 m a.s.l.; WGS84), municipality of Wenceslau Guimarães, State of Bahia, Brazil, on December 14th 2011, by MTR, RSR, Mauro Teixeira Junior and FDV.

**Etymology**. **—** The specific epithet is an adjective from Latin meaning “great” or “large” (Lewis & Short, 1891), and refers to the large size of the adult males collected from this species, which are among the largest known in the genus.

**Diagnosis**. **—** A large species (SVL 36.4 to 41.1 mm in males, *n* = 3) characterized by (1) snout mucronate in dorsal view, acute in profile; (2) mandible with two anterior large odontoids on each side in adults; (3) adults males with dorsum of body and limbs immaculate, uniformly pale yellow; (4) dorsal skin granular; (5) ventral skin cream and evenly granular, lacking distinct rows of tubercles; (6) a row of tubercles along lateral surface of forearm and tarsus; (7) tubercle subarticular from second segment of finger IV single and rounded shape; (8) canthus rostralis immaculate; (9) tympanum size corresponding to 6.6–7.1% of SVL; (10) advertisement call consisting of a series of 7–31 composite notes (half pulsed/pulsatile–half harmonic).

**Description of holotype**. **—** An adult male in good state of preservation, with a piece of muscle removed from the right thigh for molecular analyses. Raw measurements are detailed in Table S1. Body robust; head wider than long (HL 89.4% of HW; HW 39.0% of SVL; HL 34.9% of SVL); snout mucronate in dorsal view and acute in profile (Figs. 1, 2A and 2B); nostrils small, elliptical, directed anterolaterally, nearer to the tip of snout than to eye; *canthus rostralis* slightly concave; loreal region oblique, slightly concave; internarial distance smaller than eye–nostril distance (IND 67.5% of END), eyes large (ED 27.6% of HL; 24.6% of HW; 87.5% of END), prominent, situated laterally, directed anterolaterally; tympanum evident, relatively medium (TD 7.1% of SVL and TD 20.5% of HL), nearly circular, separated from posterior border of eye by approximately the half of diameter of tympanum; tympanum diameter smaller than eye to nostril distance (TD 65% of END), eye diameter (TD 74.3% of ED), interorbital distance (TD 31.3% of IOD); slightly smaller than internarial distance (TD 96.3% of IND), its diameter larger than width of discs on third finger (TD/DF3 = 1.24) and of fourth toe (TD/4TD = 1.36); tympanic annulus evident; supratympanic fold developed, covering dorsal edge of tympanum nearly straight, posteriorly slightly curved downwards and extending until near the insertion of arm; vocal sac single, subgular, poorly developed; vomerine teeth in two patches, oblique and barely separated, positioned below and between choanae; each side of mandible with two large anterior and smaller, discrete and subequal odontoids, pupil horizontal with distinct meniscus.

Arms slightly thicker than forearms (forearm/arm = 94.5%); lateral margin of forearms crenate, with four outer tubercles, those closest to hand, smaller and less evident; hand large (Fig. 2C), 31.6% of SVL, slightly crenate laterally; fingers, in the following order of length, I<II ≅IV<III; subarticular tubercles rounded, the second of the fourth finger single (Fig. S1A); a few small supranumerary tubercles; palmar tubercle well developed, rounded; thenar tubercle large, elliptical; adhesive discs developed; first finger’s disc smaller, than the others; diameter of disc of third finger equivalent to 60.0% of eye diameter; fingers fringed; webbing formula I –II 2 –3^+^ III 3 –21/2 IV.

Hind limbs long; tibia slightly longer than thigh (THL 95.6% of TBL); sum of tibia and thigh lengths 96.7% of SVL; a row of evident tubercles along lateral surface of tarsus; tarsus length smaller than foot length (TAL 65.1% of FL); foot length smaller than thigh and tibia lengths (FL 84.9% of THL; FL 81.1% of TBL); plantar surface with supernumerary tubercles; subarticular tubercles rounded; inner metatarsal tubercle well developed elongated, projecting laterally; outer metatarsal tubercle small and rounded; discs on toe I slightly smaller than those of other toes; toes fringed; webbing formula I 2 –31/2 II 2^−^ –3 ^+^ III 2^−^ –3 ^+^ IV 3^−^ –11/2 V.

Dorsal surface of body, forearms, and tibia very granular; belly granular without distinct rows of tubercles; throat, anterior and posterior surfaces of thighs and ventral surface of shanks smooth; ventral surface of thighs granular with distinct round tubercles of which a pair near thigh insertion is more prominent.

Dorsum immaculate, uniformly pale yellow; ventral surface of body, limbs and thighs cream white; iris yellow with black horizontal band projecting from pupil.

**Variation**. **—** The measurements of the specimens from the type series are summarized in Table S1. Overall, the type series is morphologically congruent with the holotype. However, one paratype (MZUESC 18265) shows the following differences: the snout outline is more rounded in dorsal and profile view, because the apical tubercle is less developed and the upper jaw more protruding; in life, the dorsum, the throat and the ventral surfaces of limbs are orange-yellow and the belly is golden-yellow (Figs. 3B and 3D); webbing formula for the left hand is I –II 2^−^ –3^+^ III 3 –2^+^ IV, and for the left foot is I 2^−^ –3 II 11/2 –3^−^ III 11/2 –3^−^ IV 21/2 –1 ^+^ V. The juvenile (MZUSP 157526) has black spots on dorsum, which are absent on adults. This suggests that this species passes through ontogenetic pattern change.

**Comparisons with congeners.—** *Phyllodytes magnus* is promptly distinguished from most of other congeners by the larger size of its males (36.4–41.1 mm vs. 15.6–28.7 mm), except for *P. maculosus* (39.7–43.5 mm) and *P. kautskyi* (38.0–42.0 mm). The single and rounded shape of the subarticular tubercle in the second segment of finger IV distinguishes *P. magnus* from *P*. *kautskyi* and *P*. *maculosus* (tubercles elongated and bifid in these species –Fig. S1). In addition, the dorsal skin is granular in *P*. *magnus* and smooth or shagreened in *P. maculosus* and *P. kautskyi* (Fig. S2).

The absence of dorsolateral dark stripes distinguishes *Phyllodytes magnus* from *P. amadoi*, *P. kautskyi* (Fig. 3F), *P. luteolus*, *P. maculosus* (Fig. 3E), *P. melanomystax*, *P. praeceptor*, *P. punctatus*, *P. tuberculosus* and *P*. *wuchereri* (all have a pair of dorsolateral dark stripes extending from the posterior corner of the eyes towards the inguinal region, though the length and density of the stripes varies according to the species). The immaculate dorsum of body and limbs distinguishes *Phyllodytes magnus* from *P. amadoi* and *P. praeceptor* (small, irregular brown patches), *P. gyrinaethes* (dorsal surfaces of body and limbs with marbled pattern), *P. maculosus* (cream colored dorsum with anastomosed brown blotches), *P*. *melanomystax* (black round spots in the dorsum, not present in all individuals/populations), *P. punctatus* and *P. tuberculosus* (distinctive brown dots on dorsum) and *P. wuchereri* (a pair of dorsolateral longitudinal white stripes and, in some individuals/populations, dorsum of hind limbs and central area of dorsum of body mottled brown).

*Phyllodytes magnus* like *P. gyrinaethes*, *P. kautskyi*, *P. maculosu* s and *P. melanomystax,* has an evenly granular venter without the distinctive rows of ventral tubercles present in *P*. *acuminatus*, *P*. *amadoi*, *P*. *brevirostris*, *P*. *edelmoi*, *P*. *luteolus*, *P*. *megatympanum*, *P*. *punctatus*, *P. praeceptor*, *P*. *tuberculosus* and *P*. *wuchereri.* The immaculate *canthus rostralis* and snout also distinguishes *P. magnus* from *P*. *melanomystax* (thick dark brown stripe on the snout and *canthus rostralis*). Furthermore, the absence of highlighted color in groin also distinguishes *P. magnus* from *P. megatympanum* (yellow groin) and *P. gryrinaethes* (red groin). In addition, the relative tympanum size corresponding to 6.7–7.1% of SVL distinguishes *P. magnus* from *P*. *megatympanum* and *P*. *acuminatus* (TD 7.73–8.16% of SVL).

**Figure 4:**
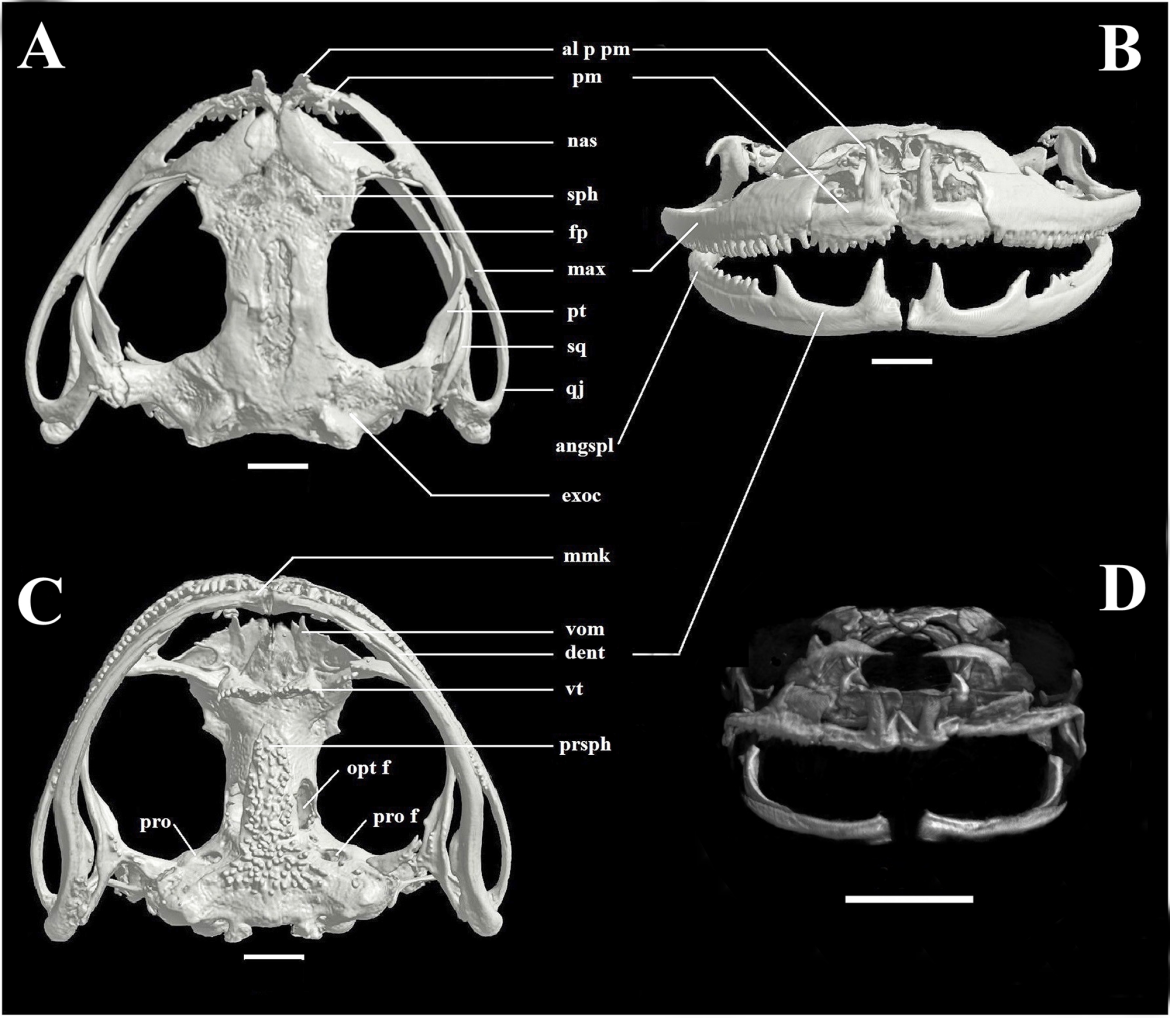
Skull of *Phyllodytes magnus* sp. nov. (A - dorsal, B - ventral and C - front view) = Adult (MZUSP 157524) and (D - front view) = juvenile (MZUSP 157526); Scale bar = two mm. Abbreviations: al p pm, alary process of premaxilla; angspl, angulosplenial; dent, dentary; exo, exoccipital; fp, frontoparietal; max, maxilla; mmk, mentomeckelian bone; nas, nasal; opt f, optic foramen; pm, premaxilla; pro, prootic; pro f, prootic foramen; prsph, parasphenoid; pt, pterygoid; qj, quadratojugal; sph, sphenethmoid; sq, squamosal; vom, vomer; vt, vomerine teeth.

**Cranial osteology**. **—** Dermal roofing bones (Fig. 4A). Skull is broad and relatively flat, wider than long. In dorsal view, nasals paired, trapezoidal, overlying the olfactory region of the skull. Sphenethmoid diamond-shaped, fused anteriorly, with posteromedial margins of nasals, and posteriorly, with anterior margins of frontoparietals. Frontoparietals present, paired, longer than wide, wider anteriorly, constricted medially. The anterior terminus of the pterygoid appears at the first third of the orbit.

Maxillary arch (Fig. 4B). Premaxillae paired, anteromedial, longer than high, with a series of 10 teeth, at the anteriormost segments of each maxillary arch, contacting laterally lower part of maxilla. Alary process of premaxilla paired, oriented dorsally, well developed, twice longer than correspondent dentary process. Maxilla paired, longer than premaxillaries, longer than high, higher anteriorly, with a series of approximately 36 teeth, decreasing in size posteriorly. Quadratojugals are positioned posterior to the maxillae and articulates with the angulosplenial.

In ventral view (Fig. 4C), paired vomers, dentigerous and associated with the anterior margins of the sphenethmoid in the palate. The parasphenoid extends posteriorly as a T-shaped ornamented bone.

Lower jaw (Fig. 4B) with paired mentomeckelian bones, dentaries and angulosplenials. Mentomeckelian bone with a very developed fanglike odontoid, oriented dorsally, slightly smaller than the allary process of premaxilla. A second well developed odontoid occurs in the dentary, posteriorly oriented, no dentition between these two projections. Angulosplenial with a thin row of five odontoids, similar to the correspondent part of maxilla. Odontoids are absent in the juvenile (Fig. 4D).

**Figure 5:**
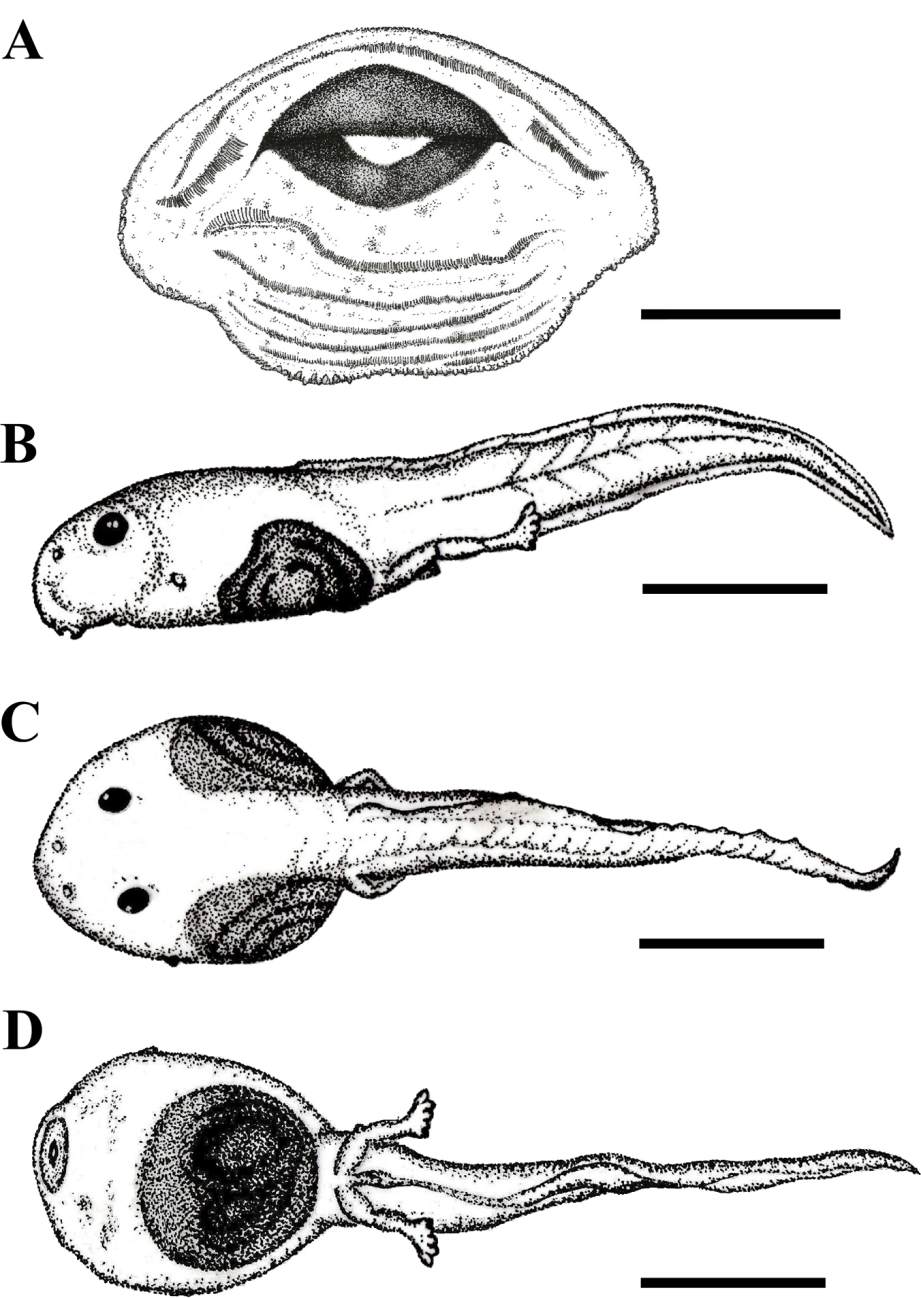
Tadpole of *Phyllodytes magnus* sp. nov. Tadpole at Gosner stage 35 (MZUSP 157525) from Wenceslau Guimarães municipality, State of Bahia, Brazil. (A) oral apparatus, (B) lateral, (C) dorsal and (D) ventral views. Scale bar = one mm (A) and five mm (B, C and D).

**Tadpole description**. **—** (Fig. 5). Body depressed (wider than high), ovoid in all views, with maximum height and width at the middle third of the body; body length corresponding to approximately 36.1% of total length. Snout uniformly rounded in all views. Eyes dorsally positioned and dorsolaterally directed; eye diameter representing 13.4% of body length. Narial apertures rounded, anterolaterally directed, closer to snout than the eyes; internarial distance representing 26.7% of body width. Spiracle single, sinistral, at the middle third of the body length and below the midline of the body height, directed posterodorsally, with a circular opening, inner wall present as slight ridge, fused to body. Vent tube central, attached to ventral fin, with opening facing the right side.

Oral apparatus (Fig. 5A) in anteroventral position, non-emarginated, surrounded by a single row of small marginal papillae, with an anterior gap on the upper labia. Labial tooth row formula (LTRF) is 2(2)/6, A1 shorter than A2. P1 and P2 of equal size, slightly longer than P3–P6. Jaw sheaths pigmented, with finely serrated edges. Upper jaw sheath arc-shaped and lower jaw sheath U-shaped.

Tail musculature height represents 50% of body height and tapers towards the tip of the tail; myomeres slightly visible in lateral view and clearly visible in dorsal view along the entire length of tail. Dorsal and ventral fins approximately equal in size, dorsal fin begins on body and continues to the tip of the tail, ventral fin begins at the end of the body and continues towards the tip of the tail, which is slightly pointed. The lateral line system is not visible. Measurements are provided in Table S2.

In formalin, the body is translucent, with brown spots distributed on dorsum, ventral and lateral regions. The intestine is visible in all surfaces. The tail is also translucent, with brown spots distributed over the surface.

Variation.**—** In stage 27, body length corresponding to 36.4% of total length, eye diameter representing 12% of body length, internarial distance representing 27.2% of body width. The LTRF is still not developed with only five posterior tooth rows, the marginal papillae appear in same quantity. Tail musculature height represents 47% of body height and tapers towards the tip of the tail. The maximum tail height is reached in stage 27 (Table S2).

**Tadpole comparisons with congeners**. **—** Nine of the 14 species of *Phyllodytes* have described tadpoles (Table S3). Like all their congenerics, tadpoles of *P. magnus* have a depressed body (wider than high). Furthermore they have no constriction, a condition similar to *P. acuminatus*, *P. brevirostris*, *P. edelmoi*, *P. melanomystax* and *P. wuchereri*, which contrast to *P. gyrinaethes* which shows anterior and lateral body constrictions and *P. luteolus, P. praeceptor* and *P. tuberculosus* which show lateral body constrictions. The LTRF of *P. magnus* is similar to *P. brevirostris* and *P. edelmoi* and differs from the remaining species that show a LTRF 2(2)/3 or 2(2)/4. The most differentiated LTRF appears to occur in *P. praeceptor* (LTRF 1/2) and in *P. gyrinaethes* (LTRF 1(1)/5). The single row of marginal papillae on the oral apparatus of *P. magnus* is similar to *P. gyrinaethes*, *P. luteolus*, *P. melanomystax*, *P. tuberculosus* and *P. wuchereri*, and differs from the remaining tadpoles that show at least two posterior rows of marginal papillae.

The dorsal fin of *P. magnus* originating on body is similar to that of *P. brevirostris* and *P. edelmoi* and differs from *P. acuminatus*, *P. gyrinaethes, P. praeceptor* and *P. wuchereri* (origin at body-tail junction) and from *P. luteolus*, *P. melanomystax* and *P. tuberculosus* (origin at tail musculature). The ventral fin originating on body is similar to all species of *Phyllodytes* except *P. melanomystax* (at the tail musculature).

The spiracle position of *P. magnus*, at midbody, in lower half is similar to those of *P. acuminatus* and *P. wuchereri*, but differs from *P. brevirostris*, *P. luteolus*, *P. melanomystax* and *P. tuberculosus* (at midbody, at midline) and from *P. edelmoi, P. praeceptor* and *P. gyrinaethes* (at body’s last third, in lower half). The eye dorsally positioned is similar to *P. acuminatus*, *P. brevirostris*, *P. luteolus*, *P. tuberculosus* and *P. wuchereri*, in contrast to *P. edelmoi, P. praeceptor* and *P. melanomystax* (dorso-laterally) and *P. gyrinaethes* (laterally).

**Figure 6:**
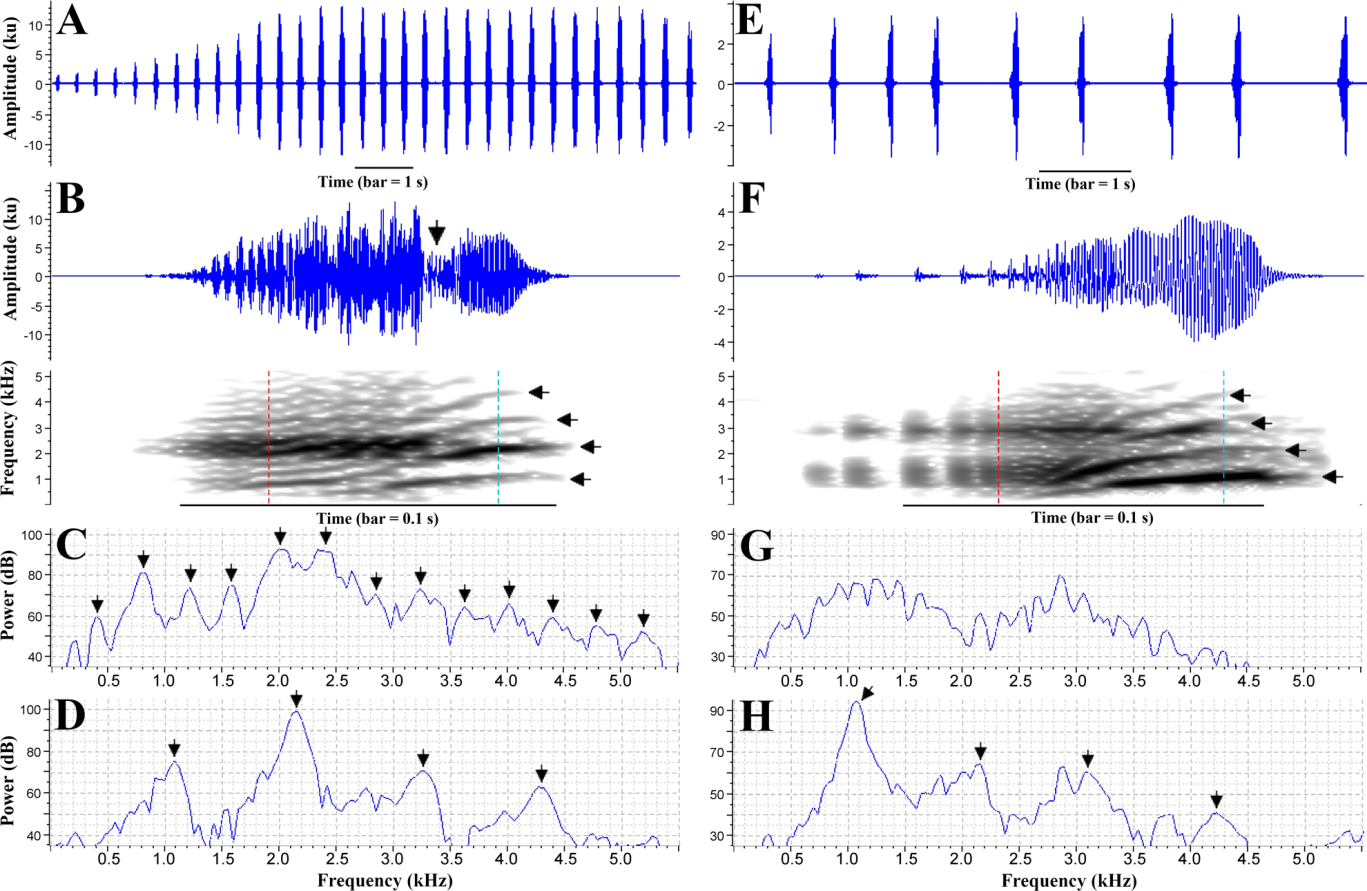
Advertisement call of *Phyllodytes magnus* sp. nov. Graphic representations of the advertisement call of the paratype MZUESC 18265 (A–D) and of the holotype MZUESC 18264 (E–H) of *Phyllodytes magnus* sp. nov. A and E: waveform of an entire call. B and F: waveform and spectrogram of one note; vertical arrow in waveform in B indicates the constriction of the waveform (see text for explanation); horizontal arrows in the spectrogram indicate the harmonics; vertical dashed lines in the spectrograms indicate the position of the spectrogram slices. C and G: spectrogram slice of the first part of the note (indicated by the left vertical line) in B and F, respectively; arrows in C indicate the sidebands, multiples of ∼0.4 kHz (see text for explanation). D and H: spectrogram slice of the second part of the note (indicated by the right vertical line) in B and F, respectively; arrows indicate the harmonics, multiples of ∼1.07 kHz (see text for explanation). Spectrographic views settings: window type: Hann, window size: 512 samples, 3 dB filter bandwidth: 124 Hz, time grid overlap: 90%, time grid size: 51 samples, frequency grid DFT size: 512 samples, frequency grid spacing: 86.1 Hz. Call voucher –FNJV 40997 (holotype) and FNJV 41382-41384 (MZUESC 18265).

**Bioacoustics results**.—Quantitative measurements of call parameters and comparisons are summarized in Tables 1 and 2. Like most *Phyllodytes* species, the advertisement call of *P. magnus* is composed by a series of notes of the same type (Fig. 6). Calls are emitted infrequently, separated by up to seven minutes of silence (Table 1). The note begins with a series of well-spaced low-amplitude pulses, which gradually raise in amplitude and emission rate until fusing into the main body of the note (Fig. 6). In the second half of the note, it changes into a sparse-harmonic sound with at least four harmonics and ascending frequency modulation (Fig. 6). The fundamental frequency is also the dominant (Table 1). The amplitude is ordinarily ascendant along the note length, reaching its maximum in the second part, near the note ending (note shape always higher than 0.5, Table 1).

**Table 1:** Quantitative measurements of the advertisement call and of the putative agonistic call (“call type II”) of *Phyllodytes magnus* sp. nov. Data is presented as mean ± standard deviation (min–max). See text for explanation of measured parameters. –: not measured, see text for explanation. ***n*** of advertisement calls (for the first three parameters) is 48 and 6 for MZUESC 18264 and MZUESC 18265, respectively. *n* of notes of advertisement calls (for the other parameters) is 91 and 63 for MZUESC 18264 and MZUESC 18265, respectively. For call type II, *n* of calls is eight and *n* of notes is 24.

	**MZUESC 18264 (holotype)**	**MZUESC 18265 (paratype)**	
**Acoustic parameters**	**Advertisement call**		**Call type II**
Call duration (s)	5.95 ± 0.74 (3.85–7.62)	9.60 ± 1.27(7.54–10.98)	0.92 ± 0.80 (0.14–2.09)
Call interval (s)	85.61 ± 55.59 (51.51–430.25)	–	–
Notes per call	9.33 ± 1.00 (7–11)	27.30 ± 4.08 (20–31)	3 ± 2.14 (1–6)
Note duration (s)	0.096 ± 0.012 (0.055–0.125)	0.086 ± 0.012 (0.056–0.101)	0.114 ± 0.05 (0.052–0.241)
Duration 90% (s)	0.044 ± 0.005 (0.027–0.054)	0.056 ± 0.012 (0.021–0.068)	0.079 ± 0.041 (0.028–0.199)
Interval between notes (s)	0.615 ± 0.146 (0.181–0.960)	0.278 ± 0.018 (0.233–0.344)	0.281 ± 0.052 (0.209–0.379)
Note emission rate (note/s)	1.42 ± 0.40 (0.94–3.61)	2.75 ± 0.15 (2.29–3.16)	2.54 ± 0.39 (1.85–3.38)
Note shape	0.77 ± 0.05 (0.67–0.92)	0.52 ± 0.13 (0.26–0.81)	0.36 ± 0.21 (0.01–0.73)
Dominant frequency (kHz)	1.03 ± 0.04 (0.86–1.29)	0.82 ± 0.06 (0.78–0.86) /2.15 ± 0.10 (1.85–2.37)	1.12 ± 0.53 (0.43–2.15)
Frequency 5% (kHz)	0.88 ± 0.3 (0.78–0.95)	1.65 ± 0.40 (0.73–1.89)	0.72 ± 0.27 (0.47–1.89)
Frequency 95% (kHz)	1.73 ± 0.19 (1.51–2.76)	2.47 ± 0.10 (2.20–2.63)	2.02 ± 0.33 (1.51–2.50)
Bandwidth 90% (kHz)	0.85 ± 0.19 (0.69–1.81)	0.82 ± 0.37 (0.52–1.68)	1.30 ± 0.32 (0.47–1.89)

**Table 2:** Acoustic parameters from the advertisement calls of ***Phyllodytes*species**. When available, data is presented as mean ± standard deviation (min–max). When the dominant frequency varies between two or more bands, the values of the different bands are separated by “/”. A sole em dash (—) means data is not available or not applicable. Two adjacent bars (//) indicates an upward modulation from the frequency at the left to the frequency at the right of the bars.

Species	Call duration (s)	Inter-calls interval (s)	Notes per call	Note duration (s)	Inter-notes interval (s)	Dominant frequency (kHz)	1st harmonic (kHz)	Presents Harmonics?	Reference
*Phyllodytes magnus* sp. nov.	6.36 ± 1.41 (3.85–10.98)	85.61 ± 55.59(51.51–430.25)	11.33 ± 5.92 (7–31)	0.092 ± 0.013 (0.055–0.125)	0.470 ± 0.200(0.181–0.960)	1.47 ± 0.56(0.78–2.37)	0.51 ± 0.05 (0.39–0.60) // 1.08 ± 0.04 (0.95–1.16)	Yes	This paper
*P. amadoi*	3.41 ± 0.28(2.99–4.11)	53.4 ± 8.5(41–68)	14.5 ± 1(13–17)	0.043 ± 0.021(0.008–0.119)	0.204 ± 0.02(0.137–0.285)	3.76 ± 0.40(2.41–4.31)	—	No	Vörös, Dias & Solé (2017)
*P. acuminatus*	—	18.99 ± 11.18 (6.03–53.4)	1–4	0.10 ± 0.03(0.03–0.17)	0.35 ± 0.05 (0.27–0.47)	2.14 ± 0.09 (2.07–2.33) / 4.28 ± 0.15 (4.05–4.57)	2.14 ± 0.09 (2.07–2.33)	Yes	Campos et al. (2014)
*P. edelmoi*	5.2 ± 0.44 (4.28–5.73)	48.27 ± 8.33 (38.11–64.83)	26.46 ± 2.3 (22–29)	0.1 ± 0.003 (0.04–0.16)	—	2.84 ± 0.16(1.49–3.32)	—	No	Lima, Lingnau & Skuk (2008)
*P. gyrinaethes*	1.7 ± 0.3(1.3–2.3)	52.4 ± 25.7 (21.2–88.7)	4.9 ± 0.6(4–6)	0.04 ± 0.01 (0.02–0.07)	0.4 ± 0.03(0.3–0.5)	2.75 ± 0.16(2.53–3.09)	—	No	Roberto & Ávila (2013)
*P. kautskyi*	3.55 ± 0.19	46.66 ± 11.45	21	0.085 ± 0.012	0.066–0.120	1.37(0.87 // 1.81)	1.37(0.87 // 1.81)	Yes	Simon & Gasparini (2003)
*P. luteolus*	5	—	8–15	0.125	—	2–6^*^	—	No	Weygoldt (1981)
*P. megatympanum*	5.91 ± 4.56 (3.2–23.63)	30.9 ± 10.31 (6.96–47.58)	13.37 ± 2.56 (10–19)	0.092 ± 0.08 (0.009–0.245)	0.305 ± 0.1(0.1–0.62)	3.98 ± 0.136 (3.56–4.12)	1.98	Yes	Marciano-Jr, Lantyer-Silva & Solé (2017)
*P. melanomystax*	0.07 ± 0.04	28.81 ± 12.48 (11.18–54.29)	1	0.07 ± 0.04	28.81 ± 12.48 (11.18–54.29)	1.39 ± 0.05 / 3.11 ± 0.25	1.39 ± 0.05	Yes	Nunes, Santiago & Juncá (2007)
*P. praeceptor*	5.34 ± 1.53(3.02–9.41)	48.5 ± 21.2(29.5–99.2)	8.39 ± 1.55(6–12)	0.295 ± 0.075(0.141–0.538)	0.389 ± 0.102(0.236–1.057)	3.05 ± 0.12(2.93–3.27)	—	No	Orrico, Dias & Marciano-Jr (2018)
*P. tuberculosus*	6.72 ± 1.73 (4.65–9.35)	—	18.60 ± 3.36 (14–23)	0.167 ± 0.047 (0.068–0.246)	0.214 ± 0.048 (0.067–0.357)	2.46 ± 0.45 (1.68–3.27)	—	No	Juncá et al. (2012)
*P. wuchereri*	2.8–6.8	134 ± 10 (120–143)	10–21	0.05–0.32	0.09–0.21	1.12–1.46 / 2.67–3.53	—	No	Cruz, Marciano & Napoli (2014) and Magalhães, Juncá & Garda (2015)

**Notes.**

* These values, separated by en dash, are not minimum and maximum in the statistical sense, instead they represent approximately the minimum and maximum of a frequency bandwidth.

The advertisement call of the paratype MZUESC 18265 has some differences in comparison to the holotype’s call (Table 1). It has more notes per call and higher note emission rate. It also has two discernible parts but, in this case, the first part is much more conspicuous and it has a faster rise of amplitude, which is marked by intense modulations that give a “noisy” (pulsatile) aspect to the waveform and produces many sidebands in the spectrogram (multiples of ∼0.4 kHz) (Fig. 6). The transition from the first to the second part of the note is well marked by a constriction in the waveform, i.e., an abrupt decay followed by a subsequent rise of amplitude (Fig. 6). The note shape is way more variable than that of the holotype (Table 1) because the maximum amplitude is reached sometimes near the beginning of note and sometimes near its ending.

We believe the differences between the calls of the two recorded individuals are due to their different levels of motivation or due to geographic variation. Further studies should address the variation in advertisement calls of this species.

A second type of call was emitted exclusively in response to our imitations, suggesting an agonistic function. This call is composed by a single note or a series of up to six notes with decreasing duration. The first notes (“A notes”) have conspicuous secondary amplitude modulation, giving a “multipulsed” fashion to the waveform with up to five amplitude peaks (“pulses”) (Fig. S3). The spectrogram of this type of note has harmonics and fast A-shaped frequency modulations (Fig. S3). The last notes of the series (“B notes”) resemble the notes of the advertisement call. This type of call was emitted with much lower amplitude, only about one tenth of the maximum amplitude of the advertisement call. The duration of note of call type II is slightly longer than the duration of advertisement call note, but regarding the dominant frequency there is no great difference between the two types of call (Table 1).

**Bioacoustic comparisons with congeners**. **—** The advertisement call of *Phyllodytes magnus* is unique in the genus by having composite notes with two components of approximately same duration: the first, pulsed/pulsatile, and the second, harmonic (Table 2). Though *P. melanomystax* also presents composite notes, its harmonic component is much longer than the noisy (pulsatile) component, which is present both in the beginning and in the end of the note (Fig. S4). The other species calls have either exclusively pulsed/pulsatile *(P. amadoi, P. edelmoi, P. gyrinaethes, P. luteolus, P. tuberculosus, P. praeceptor* and *P. wuchereri*) or harmonic (*P. acuminatus, P. kautskyi, P. megatympanum*) notes.

Furthermore, the dominant frequency in *Phyllodytes magnus* (0.78–2.37 kHz) is lower than in *P. amadoi*, *P. gyrinaethes*, *P. megatympanum* and *P. praeceptor* (combined dominant frequencies: 2.41–4.31 kHz), as well as the frequency of the first harmonic in *P. magnus* (0.39–1.16 kHz) is lower than in *P. acuminatus*, *P. megatympanum* and *P. melanomystax* (combined first harmonic frequencies: 1.39–2.33 kHz). The number of notes per call in *P. magnus* (7–31 notes) is higher than in *P. acuminatus* (1–4 notes), *P. gyrinaethes* (4–6 notes) and *P. melanomystax* (single-note call). The advertisement call of *Phyllodytes magnus* can be distinguished from *P. kautskyi* by lacking a downward frequency modulation at the end of the note (marked in *P. kautskyi* - see Simon & Gasparini, 2003) and by a longer interval between the notes (*P. magnus* = 0.181–0.960 s versus *P. kaustskyi* = 0.066–0.120 s).

**Figure 7:**
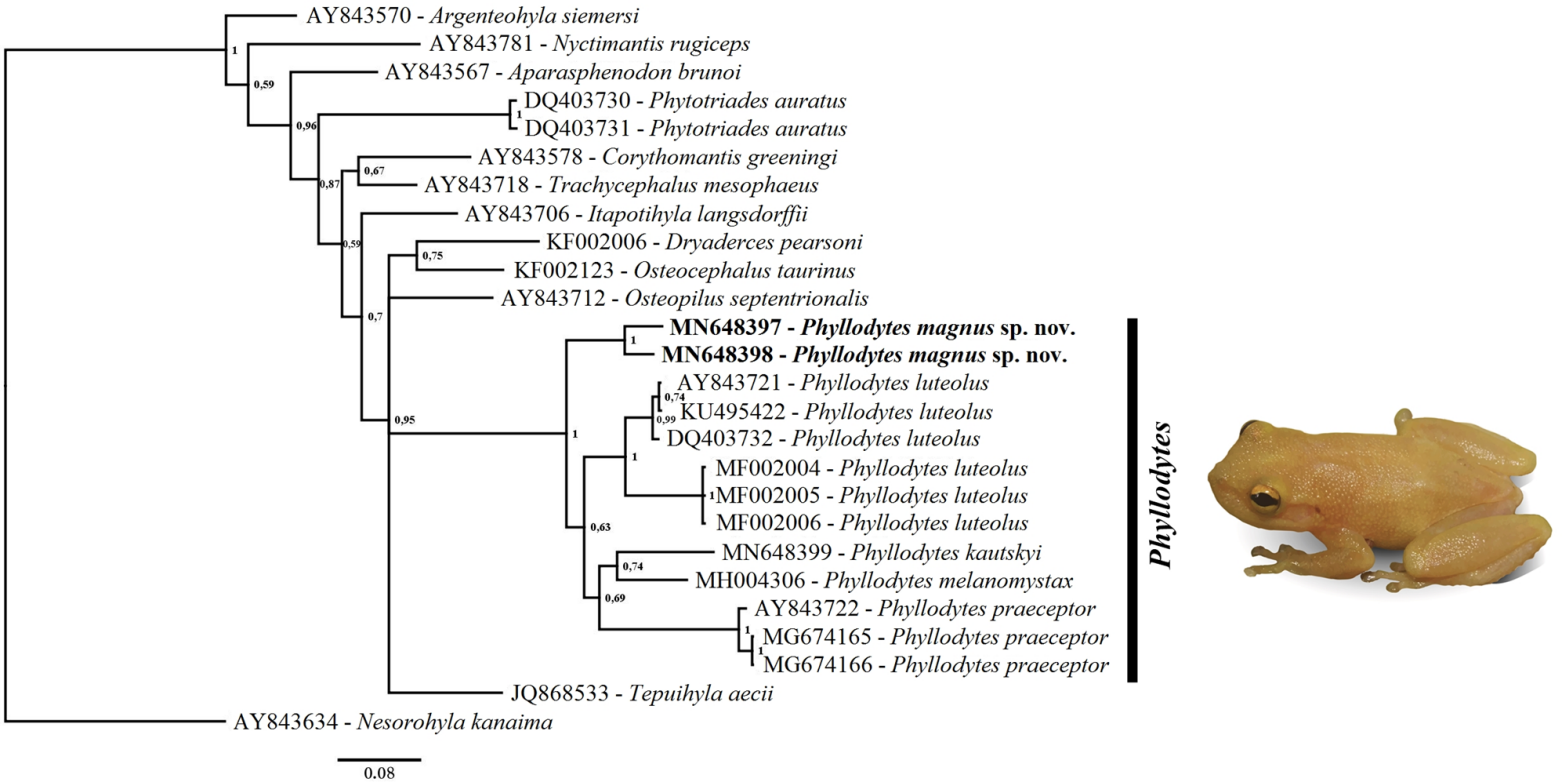
Phylogenetic relationship of genus *Phyllodytes* through 16S mitochondrial rRNA fragment gene (791 bp). Bayesian posterior probabilities values are indicated close to the branches.

**Molecular results**. **—** Our results recovered *Phyllodytes* as a well-supported monophyletic group (Fig. 7), in a polytomy together with *Tepuihyla*, *Osteopilus* and *Osteocephalus* + *Dryaderces*. The relationships of the main clades recovered within Lophyohylini received low support, as well as within *Phyllodytes*. The new species was recovered as sister to other analyzed species of *Phyllodytes* (Fig. 7). Values of genetic distance between *Phyllodytes magnus* and the other analyzed species of the genus range between 6.4 to 10.2% being lowest compared to *P. luteolus* (Table S4).

**Figure 8:**
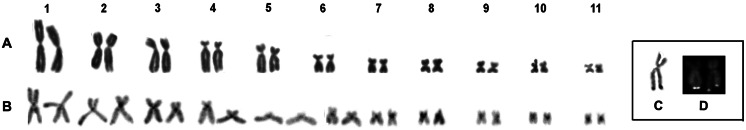
Karyotype of *Phyllodytes magnus* sp. nov. Giemsa staining (A) and C-banding (B). Highlighted are the NOR-bearing chromosomes after silver nitrate staining (C) and FISH with 18S rDNA probe (D).

**Cytogenetic results**. **—** We analyzed 35 somatic metaphases and defined the diploid number of 2*n* = 22 chromosomes and NF = 44 (Fig. 8A). Chromosome pairs 1, 7, 8, 9, 10 and 11 are metacentric, pairs 2, 3, 5 and 6 submetacentric and pair 4 subtelocentric. C banding identified a small amount of constitutive heterochromatin, as conspicuous blocks positioned pericentrically in all chromosomes. The NORs are euchromatic (Fig. 8B). The impregnation with silver nitrate showed single NORs placed terminally in the long arms of pair 2. The number and position of NORs sites were confirmed after FISH with 18S rDNA probe (Fig. 8C, Fig. 8D).

**Geographic Distribution and Natural history**. **—** *Phyllodytes magnus* is known from three localities from the Atlantic Rainforest of Bahia (Fig. S5). Calls of this species have also been heard in the municipalities of Almadina, Ilhéus, Igrapiúna and Camacan, state of Bahia, but as no vouchers or recordings were obtained, these records need to be confirmed.

Although one specimen of *Phyllodytes magnus* was in a ground bromeliad, most calling males were in canopy bromeliads. At Parque Estadual da Serra do Conduru, *Phyllodytes magnus* is syntopic with four congeners (*P. maculosus*, *P. megatympanum*, *P. melanomystax* and *P*. *praeceptor*), although apparently less abundant than its relatives. In an area of 52 ha we heard only five males in a one-night search. All were calling from epiphytic giant bromeliads of the genus *Hohenbergia* with diameter superior to 1.50 m and at heights between 3.10 m and 11.30 m (*x* = 7.72 m ± 3.56 m). We found a male of *Phyllodytes melanomystax* in the same bromeliad where we collected the paratype MZUESC 18265, though in a different axil.

## Discussion

*Phyllodytes magnus* can be readily distinguished from their congeners by morphological (large size and immaculate dorsum), acoustical (advertisement call with 7 to 31 notes; amplitude modulated notes with the first part pulsed/pulsatile and the second part sparse-harmonic) and molecular characteristics (>6% genetic distance from all analyzed congeners).

We remark for the first time the presence of composite notes (i.e., a note composed by a harmonic part plus a pulsed/pulsatile part) in the advertisement call of a *Phyllodytes* species. When comparing the calls of congeners we found that this feature occurs in *Phyllodytes melanomystax* as well. This can be observed, in the waveform, as an intense amplitude modulated (noisy) section and, in the spectrogram, as a dense and broad range of sidebands, both in the beginning and in the end of the note, while the midnote is comprised by a less noisy waveform and harmonics in the spectrogram (Fig. S4). We believe that what Nunes, Santiago & Juncá (2007) interpreted as being artifacts in the call of *P. melanomystax* may actually represent these natural components. We recommend that further call descriptions should pay attention to this feature as it seems to be an important character and can be more widespread in the genus, as well as in other frogs (e.g., *Hyla gratiosa*, see Gerhardt, 1981).

Although there are proposals to allocate *Phyllodytes* species into groups based on color pattern (Peixoto, Caramaschi & Freire, 2003; Caramaschi, Peixoto & Rodrigues, 2004) or bioacoustic characteristics (Roberto & Ávila, 2013), it has only been possible to test these proposals very recently within a more comprehensive phylogenetic context and none of them have been recovered as monophyletic (Blotto et al., 2020). Another question that hangs on *Phyllodytes* is it’s relationship with *Phytotriades*. Most of the studies did not recover this genus as sister taxon (e.g., Jowers, Downie & Cohen, 2008; Wiens et al., 2010; Pyron & Wiens, 2011; Pyron, 2014; Ron et al., 2016; Jetz & Pyron, 2018) although other authors obtained this result (Moen & Wiens, 2009; Duellman, Marion & Hedges, 2016). Even when we increase the number of analyzed species of *Phyllodytes* (previously there were only two species of this genus with sequences available for analysis) we did not recover these genera as close relatives. The same result was obtained in a phylogenetic study contemplating a larger sampling of mitochondrial and nuclear genes from more than 96% of the species of the Lophyohylini tribe and all recognized Phyllodytes species, including *P. magnus* as P. sp. 2 (Blotto et al., 2020).

*Phyllodytes magnus* presents 2*n* = 22 chromosomes and single NORs placed in the terminal region of the long arms of pair 2, the same condition described to *P. luteolus* and *P. edelmoi* (Gruber, Haddad & Kasahara, 2012) the only two species cytogenetically studied. However, *P. magnus* has a different macrochromosome arrangement. The most frequent chromosome number in Hylidae is 2*n* = 24, however, variations of this diploid number are not rare for this family (Baldissera Jr, Oliveira & Kasahara, 1993; Catroli & Kasahara, 2009). Gruber, Haddad & Kasahara (2012) proposed that the diploid number reduction observed in *Phyllodytes* should be considered as a synapomorphy for the genus, a result of a chromosomal fusion involving the nucleolar chromosomes. Our data confirmed this proposal and the few differences in the macro - (pairs 4 and 6) and microchromosome constitution (C-banding pattern) between these species are probably results of non-robertsonian rearrangements. Too few is known about cytogenetic of species of *Phyllodytes*. This lack of knowledge does not allow a more robust discussion about the patterns of chromosomal evolution in the genus.

## Conclusions

Almost 60% (nine species) of the known diversity of *Phyllodytes* can be found in the Atlantic Forest of southern Bahia, the region with the highest concentration of representatives of this genus. Over half of these species are endemic to the state: *P. wuchereri*, *P. praeceptor*, *P. megatympanum, P. amadoi* and *P. magnus*, the latter three are known from a few localities or only from the type locality (Cruz, Marciano & Napoli, 2014; Marciano-Jr, Lantyer-Silva & Solé, 2017; Vörös, Dias & Solé, 2017; this study). The high diversity of *Phyllodytes* in this part of the central corridor of the Atlantic Forest (Carnaval et al., 2009) is not unique. Other genera like *Adelophryne* (Fouquet et al., 2012; Lourenço-de Moraes et al., 2018), *Adenomera* (Fouquet et al., 2014), *Gastrotheca* (Teixeira-Jr et al., 2012) or the gymnophthalmid lizard genus *Leposoma* (Rodrigues et al., 2013), to cite a few, have their highest species diversity in this part of the biome. The high diversity of *Phyllodytes* and endemism brings support to this pattern and highlights the evolutionary importance of this area. Furthermore, together with the scarce available biological information and habitat specificity (bromeliads), it makes the Atlantic Forest of the state of Bahia a priority area for research and conservation of *Phyllodytes*.

##  Supplemental Information

10.7717/peerj.8642/supp-1 Figure S1 Comparison of subarticular tubercles from second segment of finger IV among *Phyllodytes magnus* sp. nov., *P. kautskyi* and *P. maculosus* (A) rounded and single in *Phyllodytes magnus* sp. nov. (MZUESC 18264); (B) and (C) tubercle elongated and bifid in *P. kautskyi* (MZUESC 17427) and *P. maculosus* (MZUESC 17827), respectively. Scale bar = 1 mm.

10.7717/peerj.8642/supp-2 Figure S2 Differences between dorsal skin texture: (A) smooth in *Phyllodytes kautskyi (* MZUESC 17428); (B) shagreened in *P. maculosus* (MZUESC 17433) and (C) granular in *P. magnus* sp. nov. (MZUESC 18264) Scale bar = 2 mm.

10.7717/peerj.8642/supp-3 Figure S3 Waveform (top) and spectrogram (bottom) of one call type II of *Phyllodytes magnus* sp. nov. (MZUESC 18265) The first sound in the extreme left is a vocal playback (call imitation made by the researcher) which overlaps with the beginning of the first note of the anuran call. The depicted call has only three notes. Notice the amplitude and frequency modulations in first two notes (“A notes”) and the difference compared to the last note (“B note”), which resembles a little an advertisement call note. Spectrographic views settings: window type: Hann, window size: 512 samples, 3 dB filter bandwidth: 124 Hz, time grid overlap: 90%, time grid size: 51 samples, frequency grid DFT size: 512 samples, frequency grid spacing: 86.1 Hz. Call voucher –FNJV 41384.

10.7717/peerj.8642/supp-4 Figure S4 Advertisement call of *Phyllodytes melanomystax* (A) waveform and spectrogram of a sequence of two calls (= notes). (B) waveform and spectrogram of one note; the squares highlight the pulsatile (“noisy”) components; the vertical lines indicate the position of the spectrogram slices. (C) spectrogram slice of the pulsatile component, indicated by the first vertical line in the note depicted in B; notice the nearly homogenous spectrum. D: spectrogram slice of the harmonic component, indicated by the second vertical line in the note depicted in B; notice the well defined harmonics, multiples of ∼1.8 kHz. Recorded on March 14th 2013 at the municipality of Uruçuca, state of Bahia. Temperature and air humidity unknown. Call voucher –FNJV 41385. Specimen voucher –MHNJCH 632.

10.7717/peerj.8642/supp-5 Figure S5 Geographic distribution of *Phyllodytes magnus* sp. nov This species is known from three localities from the Atlantic Rainforest of the State of Bahia: (1) Serra da Jibóia, located between the municipalities of Santa Terezinha and Elísio Medrado (star); (2) Parque Estadual da Serra do Conduru in the municipality of Uruçuca (square) and (3) Estação Ecológica Estadual de Wenceslau Guimarães, municipality of Wenceslau Guimarães (pentagon).

10.7717/peerj.8642/supp-6 Table S1 Measurements (in mm) of the type series of *Phyllodytes magnus* sp. nov Characters: SVL, snout-vent length, HW, head width, HL, head length, IND, internarial distance, END, eye-nostril distance, IOD, interorbital distance, ED, eye diameter, TD, tympanum diameter, THL, thigh length, TBL, tibia length, TAL, tarsus length, FL, foot length, HAL, hand length, DF3, finger III diameter and 4TD, toe IV disc diameter. SD, standard deviation

10.7717/peerj.8642/supp-7 Table S2 Measurements (in mm) of 13 morphometric characters of two different stages of the *Phyllodytes magnus* tadpoles (MZUSP 157525) from Municipality of Wenceslau Guimarães, State of Bahia, Brazil **** Characters: TL = total length, BL = body length, BH = body height, BW = body width, IOD = interorbital distance, IND = internarial distance, ED = eye diameter, END = eye-nare distance, NSD = nare-snout distance, TAL = tail length, MTH = maximum tail height, TMH = tail muscle height and TMW = tail muscle width.St = Stage Gosner, 1960) ****

10.7717/peerj.8642/supp-8 Table S3 External morphological characteristics of *Phyllodytes* tadpoles described in the literature and the present paper St. –Stage Gosner, 1960), TL = total length (in mm), BRL = body length relative to TL (BL/TL), LTRF = labial tooth row formula, MP = rows of marginal papillae, EOA = emargination of oral apparatus (NE = non-emarginated), BF = body format, BC = body constriction, DFO = dorsal fin origin, VFO = ventral fin origin, SP = spiracle position, EP = eye position, * - character states based on illustrations provided in the original tadpole description.

10.7717/peerj.8642/supp-9 Table S4 Uncorrected p–distances of 16S mitochondrial rRNA fragment gene (517 bp) among individuals of* Phyllodytes*

10.7717/peerj.8642/supp-10 Supplemental Information 1 New sequences (16S rRNA) of *Phyllodytes magnus* sp. nov. (MZUESC 18264 and 18265) and *P. kautskyi* (MBML 8818)

10.7717/peerj.8642/supp-11 Appemdix A1 Material examined in addition to the new species

10.7717/peerj.8642/supp-12 Supplemental Information 2 Advertisement call of *Phyllodytes magnus* sp. nov Specimen voucher = MZUESC 18264 (holotype); Call voucher = FNJV40997; March 2nd 2015; air temperature = 19 °C.

10.7717/peerj.8642/supp-13 Supplemental Information 3 Advertisement call of *Phyllodytes magnus* sp. nov Specimen voucher = MZUESC 18265 (paratype); Call voucher = FNJV 41382-41384; June 18th 2014; air temperature = 19 °C

10.7717/peerj.8642/supp-14 Supplemental Information 4 Advertisement call of *Phyllodytes magnus* sp. nov Specimen voucher = MZUESC 18265 (paratype); Call voucher = FNJV 41382-41384; June 18th 2014; air temperature = 19 °C

10.7717/peerj.8642/supp-15 Supplemental Information 5 Advertisement call of *Phyllodytes magnus* sp. nov Specimen voucher = MZUESC 18265 (paratype); Call voucher = FNJV 41382-41384; June 18th 2014; air temperature = 19 °C

10.7717/peerj.8642/supp-16 Supplemental Information 6 Advertisement call of *Phyllodytes melanomystax* Specimen voucher = MHNJCH 632; Call voucher = FNJV 41385; March 14h 2013; air temperature unknown

## Additional Information and Declarations

### DNA Deposition

The following information was supplied regarding the deposition of DNA sequences: New sequences of *Phyllodytes magnus* sp. nov. (MZUESC 18264 and 18265) and *P. kautskyi* (MBML 8818) are available at GenBank: MN648397 to MN648399. Raw sequence data is available in the Supplemental File.

### Data Availability

The following information was supplied regarding data availability: All recordings are available at the Fonoteca Neotropical Jacques Vielliard (https://www2.ib.unicamp.br/fnjv/): FNJV 40997, 41382-41384 and in the Supplemental Files. Additional data is available at Morphosource:
https://www.morphosource.org/Detail/MediaDetail/Show/media_id/47746.

### New Species Registration

The following information was supplied regarding the registration of a newly described species: Publication LSID: urn:lsid:zoobank.org:pub:FD2CACD2-F59F-4B75-947D-B576701424D9 Phyllodytes magnus LSID: urn:lsid:zoobank.org:act:D8DF9024-57C1-413B-9D05-B0CF05B7929C.
