# Supplementary figures and images for: A new large canopy-dwelling species of Phyllodytes Wagler, 1930 (Anura, Hylidae) from the Atlantic Forest of the state of Bahia, Northeastern Brazil
Source: PeerJ. 2020 Jun 23;8:e8642. doi: 10.7717/peerj.8642 (PMC7319025; doi:10.7717/peerj.8642)

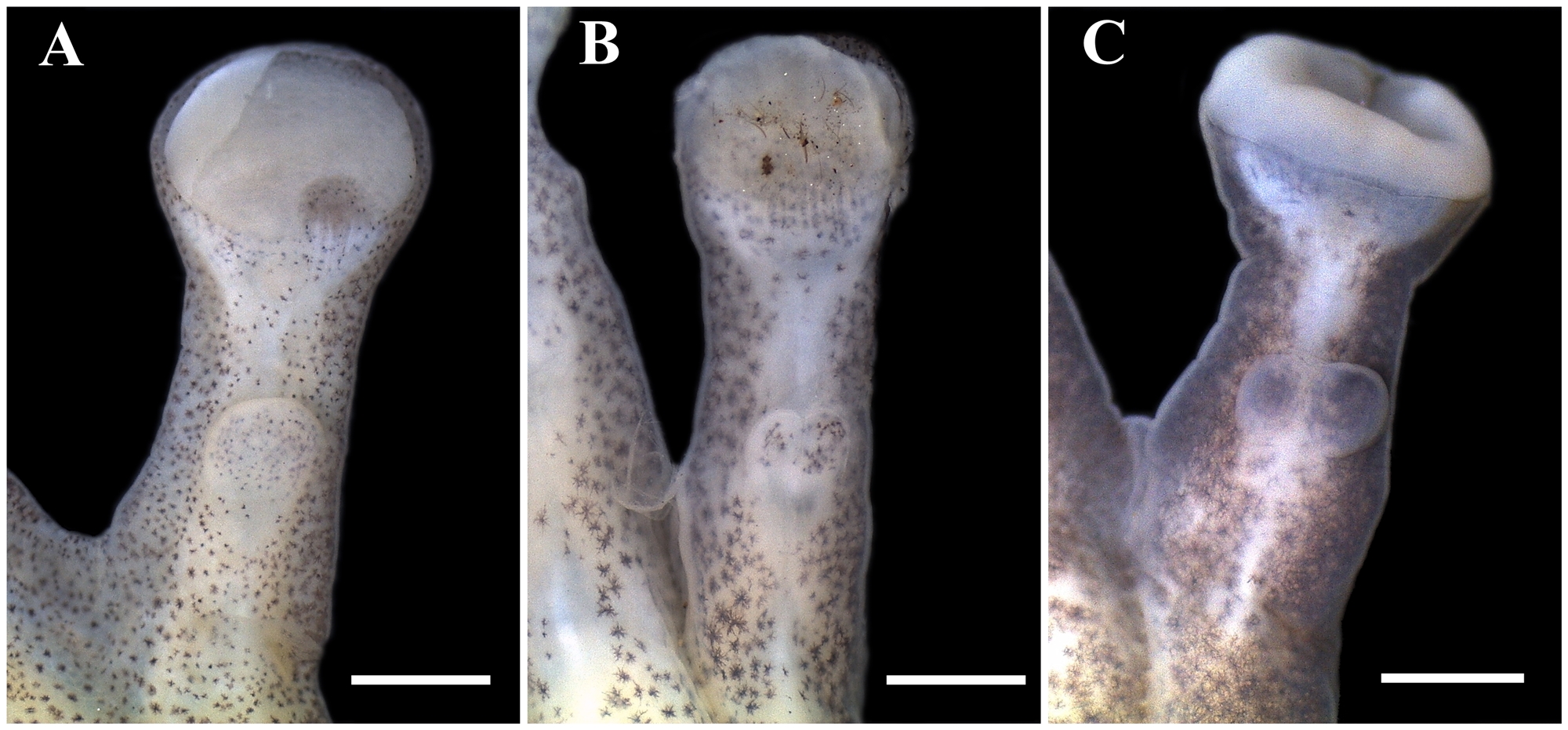

Supplement: Figure S1 — (A) rounded and single in Phyllodytes magnus sp. nov. (MZUESC 18264); (B) and (C) tubercle elongated and bifid in P. kautskyi (MZUESC 17427) and P. maculosus (MZUESC 17827), respectively. Scale bar = 1 mm. [file peerj-08-8642-s001.jpg]

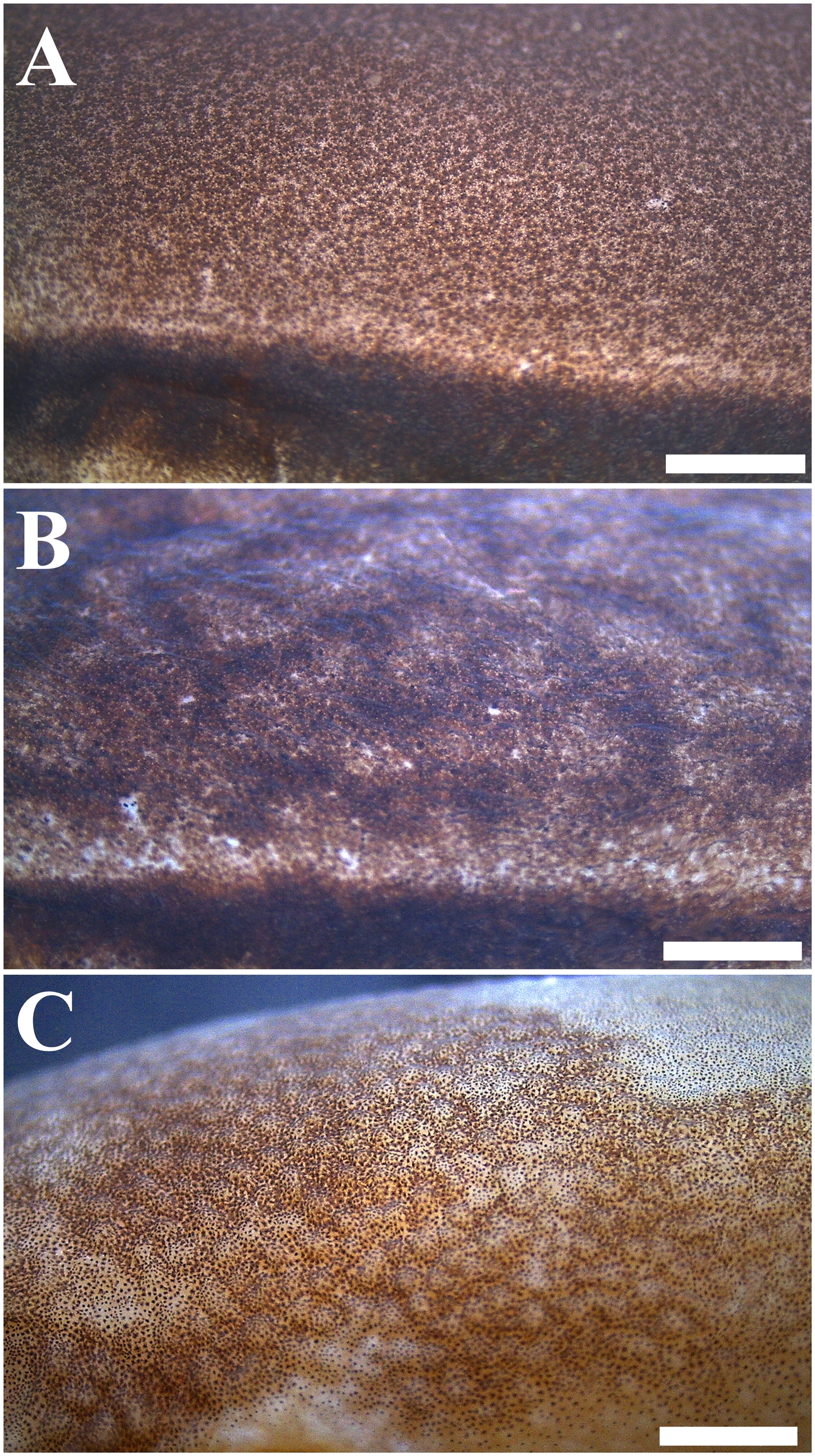

Supplement: Figure S2 — Scale bar = 2 mm. [file peerj-08-8642-s002.jpg]

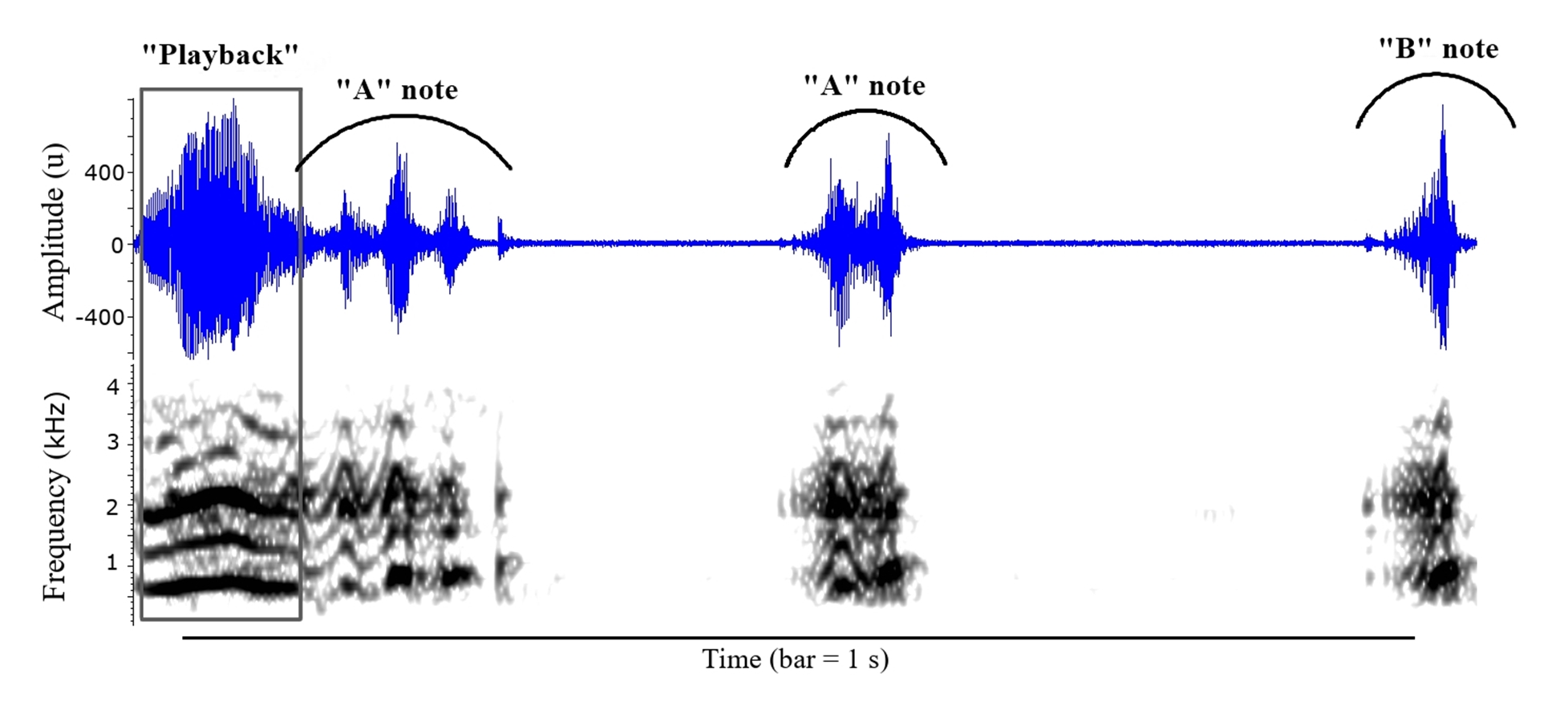

Supplement: Figure S3 — The first sound in the extreme left is a vocal playback (call imitation made by the researcher) which overlaps with the beginning of the first note of the anuran call. The depicted call has only three notes. Notice the amplitude and frequency modulations in first two notes (“A notes”) and the difference compared to the last note (“B note”), which resembles a little an advertisement call note. Spectrographic views settings: window type: Hann, window size: 512 samples, 3 dB filter bandwidth: 124 Hz, time grid overlap: 90%, time grid size: 51 samples, frequency grid DFT size: 512 samples, frequency grid spacing: 86.1 Hz. Call voucher –FNJV 41384. [file peerj-08-8642-s003.png]

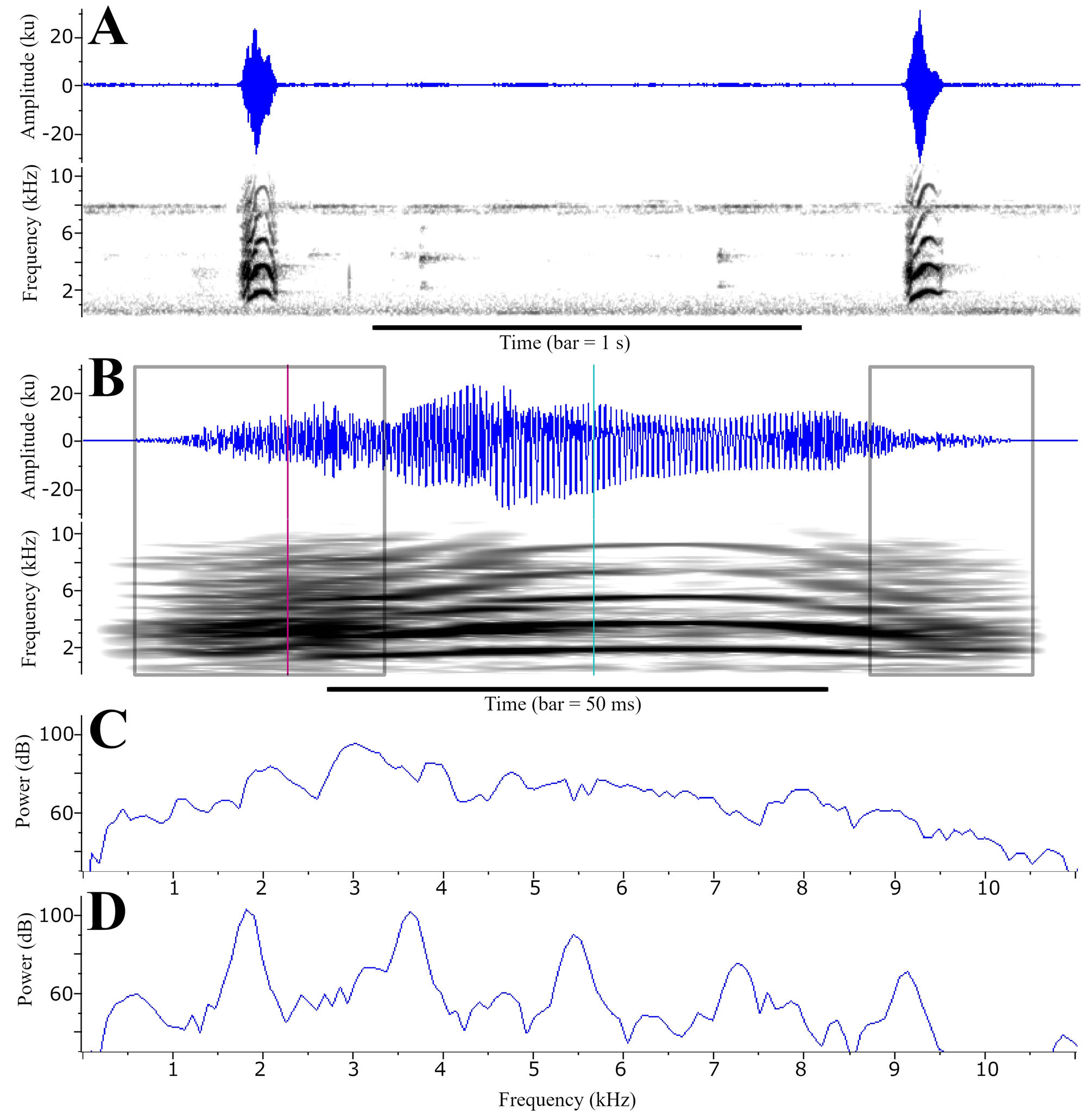

Supplement: Figure S4 — (A) waveform and spectrogram of a sequence of two calls (= notes). (B) waveform and spectrogram of one note; the squares highlight the pulsatile (“noisy”) components; the vertical lines indicate the position of the spectrogram slices. (C) spectrogram slice of the pulsatile component, indicated by the first vertical line in the note depicted in B; notice the nearly homogenous spectrum. D: spectrogram slice of the harmonic component, indicated by the second vertical line in the note depicted in B; notice the well defined harmonics, multiples of ∼1.8 kHz. Recorded on March 14th 2013 at the municipality of Uruçuca, state of Bahia. Temperature and air humidity unknown. Call voucher –FNJV 41385. Specimen voucher –MHNJCH 632. [file peerj-08-8642-s004.png]

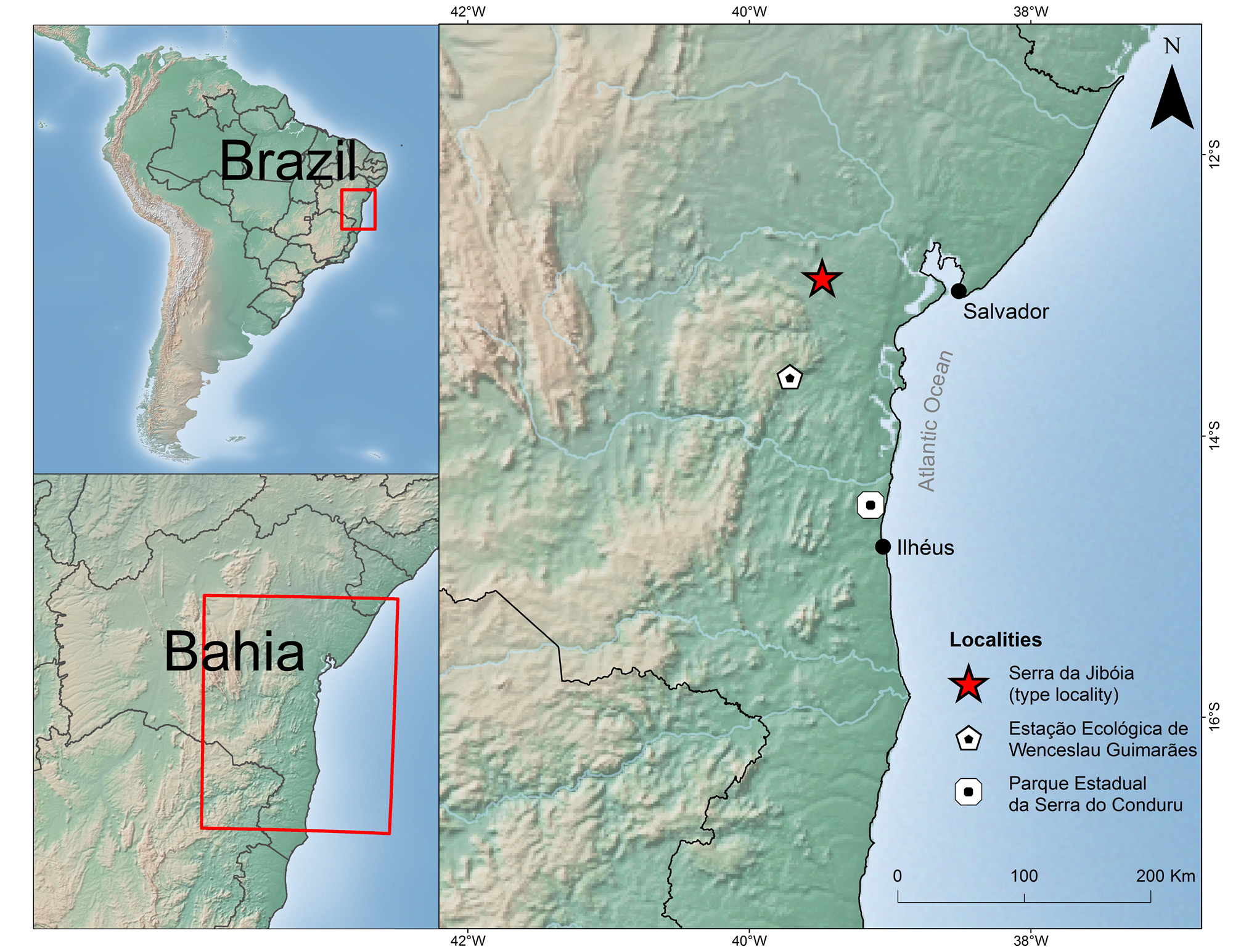

Supplement: Figure S5 — This species is known from three localities from the Atlantic Rainforest of the State of Bahia: (1) Serra da Jibóia, located between the municipalities of Santa Terezinha and Elísio Medrado (star); (2) Parque Estadual da Serra do Conduru in the municipality of Uruçuca (square) and (3) Estação Ecológica Estadual de Wenceslau Guimarães, municipality of Wenceslau Guimarães (pentagon). [file peerj-08-8642-s005.png]
