# Supplementary material for: A new large canopy-dwelling species of Phyllodytes Wagler, 1930 (Anura, Hylidae) from the Atlantic Forest of the state of Bahia, Northeastern Brazil
Source: PeerJ. 2020 Jun 23;8:e8642. doi: 10.7717/peerj.8642 (PMC7319025; doi:10.7717/peerj.8642)
Supplement: Table S1 — Characters: SVL, snout-vent length, HW, head width, HL, head length, IND, internarial distance, END, eye-nostril distance, IOD, interorbital distance, ED, eye diameter, TD, tympanum diameter, THL, thigh length, TBL, tibia length, TAL, tarsus length, FL, foot length, HAL, hand length, DF3, finger III diameter and 4TD, toe IV disc diameter. SD, standard deviation [file peerj-08-8642-s006.docx]

**Table S1:**

**Measurements (in mm) of the type series of *Phyllodytes magnus* sp. nov.**

**Characters: SVL = snout-vent length, HW = head width, HL = head length, IND = internarial distance, END = eye-nostril distance, IOD = interorbital distance, ED = eye diameter, TD = tympanum diameter, THL= thigh length, TBL = tibia length, TAL = tarsus length, FL = foot length, HAL = hand length, DF3 = finger III diameter and 4TD = toe IV disc diameter. SD = standard deviation.**

| **Characters** | **Holotype** | **Paratype** | **Paratype** | **Mean ±** **SD** | **Paratype - juvenile** |
| --- | --- | --- | --- | --- | --- |
|  | **MZUESC 18264** | **MZUESC 18265** | **MZUSP 157524** |  | **MZUSP 157526** |
| SVL | 36.4 | 40.6 | 41.1 | 39.4 ± 2.6 | 16.5 |
| HW | 14.2 | 16.8 | 17.3 | 16.1 ± 1.7 | 6.0 |
| HL | 12.7 | 14.9 | 13.2 | 13.6 ± 1.2 | 4.7 |
| IND | 2.7 | 3.1 | 2.6 | 2.8 ± 0.3 | 1.1 |
| END | 4.0 | 4.7 | 3.6 | 4.1 ± 0.6 | 1.6 |
| IOD | 8.3 | 9.4 | 9.3 | 9.0 ± 0.6 | 3.6 |
| ED | 3.5 | 3.9 | 4.2 | 3.9 ± 0.4 | 1.7 |
| TD | 2.6 | 2.7 | 2.8 | 2.7 ± 0.1 | 0.6 |
| THL | 17.2 | 19.3 | 19.4 | 18.6 ± 1.2 | 7.4 |
| TBL | 18.0 | 20.6 | 20.8 | 19.8 ± 1.6 | 7.6 |
| TAL | 9.5 | 11.4 | 10.4 | 10.4 ± 1.0 | 3.7 |
| FL | 14.6 | 15.3 | 17 | 15.6 ± 1.2 | 5.9 |
| HAL | 11.5 | 12.8 | 12.9 | 12.4 ± 0.8 | 4.6 |
| DF3 | 2.1 | 2.2 | 2.3 | 2.2 ± 0.1 | 0.7 |
| 4TD | 1.9 | 1.6 | 1.9 | 1.8 ± 0.2 | 0.6 |
