# Supplementary material for: A new large canopy-dwelling species of Phyllodytes Wagler, 1930 (Anura, Hylidae) from the Atlantic Forest of the state of Bahia, Northeastern Brazil
Source: PeerJ. 2020 Jun 23;8:e8642. doi: 10.7717/peerj.8642 (PMC7319025; doi:10.7717/peerj.8642)
Supplement: Table S2 — Characters: TL = total length, BL = body length, BH = body height, BW = body width, IOD = interorbital distance, IND = internarial distance, ED = eye diameter, END = eye-nare distance, NSD = nare-snout distance, TAL = tail length, MTH = maximum tail height, TMH = tail muscle height and TMW = tail muscle width.St = Stage Gosner, 1960) [file peerj-08-8642-s007.docx]

**Table S2:**

**Measurements (in mm) of 13 morphometric characters of two different stages of the *Phyllodytes magnus* tadpoles (MZUSP 157525) from Municipality of Wenceslau Guimarães, State of Bahia, Brazil.**

**Characters: TL = total length, BL= body length, BH = body height, BW = body width, IOD = interorbital distance, IND = internarial distance, ED = eye diameter, END = eye-nare distance, NSD = nare-snout distance, TAL = tail length, MTH = maximum tail height, TMH = tail muscle height and TMW = tail muscle width. St = Stage (Gosner 1960).**

|  | **St 35** | **St 27** |
| --- | --- | --- |
| **Character** | **(*n*=1)** | **(*n*=1)** |
| **TL** | 28.8 | 22.8 |
| **BL** | 10.4 | 8.3 |
| **BH** | 5.2 | 5.1 |
| **BW** | 7.1 | 6.6 |
| **IOD** | 3.2 | 2.7 |
| **IND** | 1.9 | 1.8 |
| **ED** | 1.4 | 1.0 |
| **END** | 1.6 | 1.1 |
| **NSD** | 0.9 | 0.6 |
| **TAL** | 18.4 | 14.5 |
| **MTH** | 3.4 | 3.7 |
| **TMH** | 2.6 | 2.4 |
| **TMW** | 2.2 | 2.1 |
