# Supplementary material for: A new large canopy-dwelling species of Phyllodytes Wagler, 1930 (Anura, Hylidae) from the Atlantic Forest of the state of Bahia, Northeastern Brazil
Source: PeerJ. 2020 Jun 23;8:e8642. doi: 10.7717/peerj.8642 (PMC7319025; doi:10.7717/peerj.8642)
Supplement: Table S3 — St. –Stage Gosner, 1960), TL = total length (in mm), BRL = body length relative to TL (BL/TL), LTRF = labial tooth row formula, MP = rows of marginal papillae, EOA = emargination of oral apparatus (NE = non-emarginated), BF = body format, BC = body constriction, DFO = dorsal fin origin, VFO = ventral fin origin, SP = spiracle position, EP = eye position, * - character states based on illustrations provided in the original tadpole description. [file peerj-08-8642-s008.docx]

**Table S3:**

**External morphological characteristics of *Phyllodytes* tadpoles described in the literature and the present paper.**

**St. – Stage (Gosner 1960), TL = total length (in mm), BRL = body length relative to TL (BL/TL), LTRF = labial tooth row formula, MP = rows of marginal papillae, EOA = emargination of oral apparatus (NE =** **non-emarginated), BF = body format, BC = body constriction, DFO = dorsal fin origin, VFO = ventral fin origin, SP = spiracle position, EP = eye position, * - character states based on illustrations provided in the original tadpole description.**

| Species | St | TL | BRL (%) | LTRF | MP  (rows) | EOA | BF | BC | DFO | VFO | SP | EP | Ref. |
| --- | --- | --- | --- | --- | --- | --- | --- | --- | --- | --- | --- | --- | --- |
| *P. acuminatus* | 38 | 26 | 41 | 2(2)/4 | two | NE | depressed | absent | body-tail junction | on body | at midbody in lower half | dorsally | *Campos et al., 2014* |
| *P. brevirostris* | 35 | 17.3–32.7 | 38 | 2(2)/5 | one anteriorly and two posteriorly | NE | depressed | absent | on body | on body | at midbody at midline | dorsally | *Vieira et al., 2009* |
| *P. edelmoi* | 28 | 27.1 | 36 | 2(2)/5–6 | two anteriorly and three posteriorly | NE | depressed | absent | on body | on body | in body's last third, in lower half | dorso-laterally | *Peixoto et al., 2003* |
| *P. gyrinaethes* | 34 | 30 | 35 | 1(1)/5 | one | NE | depressed | anteriorly and laterally | body-tail junction | on body | in body's last third, in lower half | laterally | *Peixoto et al., 2003* |
| *P. luteolus* | 36 | 29 | - | 2(2)/4 | one | NE | depressed* | laterally | at tail musculature | on body | at midbody at midline | dorsally | *Bokermann, 1966* |
| *P. magnus* | 35 | 28.8 | 36 | 2(2)/5–6 | one | NE | depressed | absent | on body | on body | at midbody in lower half | dorsally | *Present study* |
| *P. melanomystax* | 36 | 34.6 | 32 | 2(2)/3 | one | NE | depressed | absent | at tail musculature | at tail musculature | at midbody at midline | dorso-laterally | *Caramaschi et al., 1992* |
| *P. praeceptor* | 38 | 25.1 | 33.5 | 1/2 | two, with a large anterior gap | NE | depressed | slightly laterally | body-tail junction | on body | in body's last third, in lower half | dorso-laterally | *Santos et al., 2019* |
| *P. tuberculosus* | 35 | 31 | - | 2(2)/4 | one | NE | depressed* | laterally | at tail musculature | on body | at midbody at midline | dorsally | *Bokermann, 1966* |
| *P. wuchereri* | 36 | 31.5 | 38 | 2(2)/4 | one | NE | depressed | absent | body-tail junction | on body | at midbody in lower half | dorsally | *Magalhães et al., 2015* |

**References**

Bokermann, W.C.A. (1966): O gênero *Phyllodytes* Wagler, 1830 (Anura, Hylidae). *An.* Acad. Bras. Ciênc. **38**:335-344.

Campos, T.F., Lima, M.G., Nascimento, A.C., Santos, E.M. (2014): Larval morphology and advertisement call of *Phyllodytes acuminatus* Bokermann, 1966 (Anura: Hylidae) from northeastern Brazil. Zootaxa **3779**:93-100.

Caramaschi, U., Silva, H.R., Britto-Pereira, M.C. (1992): A new species of *Phyllodytes* (Anura, Hylidae) from southern Bahia, Brazil. Copeia **1992**:187-191.

Magalhães, F.D.M., Juncá, F.A., Garda, A.A. (2015): Tadpole and vocalisations of *Phyllodytes wuchereri* (Anura: Hylidae) from Bahia, Brazil. Salamandra **51**:83-90.

Peixoto, O.L., Caramaschi, U., Freire, E.M.X. (2003): Two new species of *Phyllodytes* (Anura: Hylidae) from the state of Alagoas, northeastern Brazil. Herpetologica **59**:235-246.

Santos, L.A., Costam, R.N., Solé, M., Orrico, V.G.D. (2019): The tadpole of *Phyllodytes praeceptor* (Anura: Hylidae). Zootaxa **4623**:381-386.

Vieira, W.L.S., Santana, G.G, Santos, S.C.N.C, Alves, R.R.N., Pereira-Filho, G.A. (2009): Description of the tadpoles of *Phyllodytes brevirostris* (Anura: Hylidae). Zootaxa **2119**:66-68.
