# Supplementary material for: A new large canopy-dwelling species of Phyllodytes Wagler, 1930 (Anura, Hylidae) from the Atlantic Forest of the state of Bahia, Northeastern Brazil
Source: PeerJ. 2020 Jun 23;8:e8642. doi: 10.7717/peerj.8642 (PMC7319025; doi:10.7717/peerj.8642)
Supplement: Table S4 [file peerj-08-8642-s009.docx]

**Table S4:**

**Uncorrected p–distances of 16S mitochondrial rRNA fragment gene (517 bp) among individuals of *Phyllodytes*.**

|  | **Species** | **Genbank** | **1** | **2** | **3** | **4** | **5** | **6** | **7** |
| --- | --- | --- | --- | --- | --- | --- | --- | --- | --- |
| **1** | *P. kautskyi* | MN648399 | - |  |  |  |  |  |  |
| **2** | *P. luteolus* | AY843721 | 8.0 | - |  |  |  |  |  |
| **3** | *P. luteolus* | MF002006 | 9.7 | 4.5 | - |  |  |  |  |
| **4** | *P. magnus* | MN648397 | 10.2 | 7.1 | 8.7 | - |  |  |  |
| **5** | *P. magnus* | MN648398 | 9.7 | 6.4 | 7.8 | 2.1 | - |  |  |
| **6** | *P. melanomystax* | MH004306 | 7.3 | 7.3 | 9.0 | 8.0 | 8.5 | - |  |
| **7** | *P. praeceptor* | MG674165 | 12.3 | 10.9 | 12.1 | 9.7 | 9.5 | 10.4 | - |
