# Supplementary material for: A new large canopy-dwelling species of Phyllodytes Wagler, 1930 (Anura, Hylidae) from the Atlantic Forest of the state of Bahia, Northeastern Brazil
Source: PeerJ. 2020 Jun 23;8:e8642. doi: 10.7717/peerj.8642 (PMC7319025; doi:10.7717/peerj.8642)
Supplement: Supplemental Information 1 [file peerj-08-8642-s010.docx]

>Phyllodytes_magnus_MZUESC18264_ID840_holotype_MN648397

GTAGCGGCTGCACCACTGGGATACCCTGATCCAACATCGAGGTCGTAAGCCCACTTTTTG

ATAGGGTCTCTTGAAGTGGATTGCGCTGTTATCCCTAGGGTAACTTGGTTCATTGATCAA

AATATTGGGTCAATTGATGTCAATTTATTGGTACTTAGAGTCGTGGCTCTTGGCTCAGAA

GTATATTCTTTCAGTGCGGAGGTTGAGTTTTACTCCGCGGTCGCCCCAACCTAAAACTAT

TAATCATAATATAATAATAAAGGTTTATTATTCTGGAAAAAATTTAAACATAAAATATTT

GTTACTTAGTTTGAAGCTCCATAGGGTCTTCTCGTCTTATATCTTTATCCCCGCTTCTTC

ACGGGGAGATTAGTTTCACTGATTAAAAAAAGGAGACAGTATAACCTTCGTGGTGCCATT

CATACAAGTCCCCATTTAAAGAACAATTGATTATGCTACCTTCGCGCGGTTAAGATACCG

CGGCCGTTGAATAAATCACTGGGCAGGCTGGACCTCTTATAATTACTCAAGAGGCGATGT

TTTTGGTAAACAGGCGGGGTTATTATTTGCCGAGTTCCTTCTTTTTCTTTCAATCTTTCT

TTAAATGCTCTTGTGTTAGGTTAACATAAAATTTATCAGGGTTTTCTTGGGGGGTTGCTG

ATGTAGTTTCATGAGTGTTAATTATCAGTGATGGGTTCTTGCTGATTTATACTTGCATTa

AGAGAAGGTCTACTTCTTGTTACTAGTTTTAACATTATAAATTCTATAACGGTATAGAAT

TACTCAATAAAATTAA

>Phyllodytes_magnus_MZUESC18265_MN648398

CGGCTGCACCACTGGGATACCCTGATCCAACATCGAGGTCGTAAGcCCACTTTTTGATAG

GGTCTCTTGAAGTGGATTGCGCTGTTATCCCTAGGGTAACTTGGTTCGTTGATCAAAATA

TTGGGTCAATTGATGTCAATTTATTGGTACTTAGAGTCGTGGCTCTTGGCTCAGAAGTAG

ATTCTTTCAGTGCGGAGGTTGAGTTTTACTCCGCGGTCGCCCCAACCTAAAACTATTAAT

CATAATATAACAATAAGGGTTTTTATTCTGGAAAATTTTAAACACAAAATATTTGTTACT

TAGTTTGAAGCTCCATAGGGTCTTCTCGTCTTATATTTTTATCCCCGCTTCTTCACGGGG

GGATTAGTTTCACTGATTAAAAAAAGGAGACAGTACAACCTTCGTGGTGCCATTCATACA

AGTCCCCATTTAAAGAACAATTGATTATGCTACCTTCGCGCGGTTAAGATACCGCGGCCG

TTGAATAAATCACTGGGCAGGCTGGACCTCTTATAATTTCTCAAGAGGCGATGTTTTTGG

TAAACAGGCGGGGTTATTATTTGCCGAGTTCCTTCTTTTTCTTTTAATCTTTCTTTGAAT

GCTCTCGTGTTAGGTTAACATAAAATTTATCAGGGTTTTCTTGGGGAGTTGCTAATATAA

TTTCATGGATGTTAATTATCAGTGGTGGGTTCTTGCTGATTTATACTTGCATTTAAGAGA

AGGTCTTCTTCTTGTTACTAGTTTTAACATTATAGATTCTATAATGATATAGAATTACTC

AATAAAATTAAAGGGTTCGGTTTGTTTAAAGAAATTTA

>Phyllodytes_kautskyi_MBML8818_RBF1561_MN648398

AGCGGCTGCACCACTAGGATACCCTGATCCAACATCGAGGTCGTAAGCCCACTTTTTGAT

AAGGTCTCTTGAAGTGGATTGCGCTGTTATCCCTAGGGTAACTTGGTTCGTTGATCAAAA

TATTGGGTCAACTGATGTCAATTTATTGGTGCTTAGAATAGTAATTCGTGGTTCAGAATA

GAATTCTTTCAGTGCGGAGGTTGGTTTTTACTCCGCGGTCACCCCAACCTAAAACTATTA

ATCATAATATAAAAATAGAAATTTTTATTCTGAAAATTAAAACGTGGGTAATACTTGTTA

CATAGTTTGAAGCTCCATAGGGTCTTCTCGTCTTATATTTTTATTCCCGCTTCTTCACGG

GGAGATTAGTTTCACTGATTAAAAAAAGGAGACAGTACAACCTTCGTGATGCCGTTCATA

CAAGTCCTCATTTAAAGAACAATTGATTATGCTACCTTCGCGCGGTTAAGATACCGCGGC

CGTTGAATAAATCACTGGGCAGGCTGGACCTCTTATAGGTGTTCAAAAGGCGATGTTTTT

GGTAAACAGGCGGGGTTATCATTTGCCGAGTTCCTTCTTTTTTTTTTAATCTTTCTTTTA

GTGCTCTTGTGTTAGGTTAACATAGAATTTATTAGAGTTTTCTTGAGGTGTGGTTACTAA

TATAATTTCACAGATGTTAATTATCAGTGGTGGGTCCTTGCTGATTTATACTTGCATTTA

AGAGAAGGTCTTCTTCTTGTTACTAGTTTTAACATTATAAATTCTATAATAATATAGAAT

TACTCAATAGGGTTTAA
