# Supplementary material for: A new large canopy-dwelling species of Phyllodytes Wagler, 1930 (Anura, Hylidae) from the Atlantic Forest of the state of Bahia, Northeastern Brazil
Source: PeerJ. 2020 Jun 23;8:e8642. doi: 10.7717/peerj.8642 (PMC7319025; doi:10.7717/peerj.8642)
Supplement: Appemdix A1 [file peerj-08-8642-s011.docx]

**Appendix 1.**

Additional specimens examined. For acronyms, see text.

***Phyllodytes acuminatus:* Alagoas,** Mangabeiras (MZUSP 74153 [ex-WCAB 2701] – holotype; MZUSP 74441 [ex-WCAB 2702] – paratype)

***Phyllodytes amadoi:* Bahia,** Una (MZUESC 14954 – holotype; MZUESC 14943-14945; 14952–14953; 14957; 14959 – paratypes)

***Phyllodytes kautskyi:* Espírito Santo**, Domingos Martins (MZUESC 17427–17428 – topotypes)

***Phyllodytes luteolus:* Bahia**, Caravelas (MZUESC 9888; 9891–9893); Ilhéus (CZGB 735–737; 741); **Espírito Santo,** Itaúnas (MZUESC 9782–9783)

***Phyllodytes maculosus****:* **Bahia**, Uruçuca (MZUESC 17827–17829); Porto Seguro (MZUESC 17432–17434)

***Phyllodytes megatympanum***: **Bahia**, Ilhéus (MZUESC 16061; 11671–11672 - paratopotypes)

***Phyllodytes melanomystax****:* **Bahia**, Valença (MZUSP 66102–66105 – paratypes); Ilhéus (MZUESC 598; 7300; 7502; 11669; 14245–14247); Uruçuca (MZUESC 7560)

***Phyllodytes praeceptor****:* **Bahia**, Santa Terezinha - Serra da Jibóia (MZUESC 17484 – holotype; MZUESC 17485– paratype)

***Phyllodytes punctatus***: **Sergipe**, Santo Amaro das Brotas – Gravatá (MZUSP 49899–49901; 52776–52786; 57005; 57007–57013; 57037–57038 - paratypes)

***Phyllodytes tuberculosus****:* **Bahia**, Maracás (MZUSP 74456 – holotype; 74049–74058; 74167–74166; 74457 – paratypes; MHNJCH 1120–1123 – topotypes)

***Phyllodytes wuchereri****:* **Bahia**, Camacan (MZUESC 8134; 8319; 9052; 11674).
